# Supplementary material for: Prevalence and factors associated with anaemia in children aged 6–24 months living a high malaria transmission setting in Burundi
Source: PLoS One. 2022 Sep 2;17(9):e0273651. doi: 10.1371/journal.pone.0273651 (PMC9439186; doi:10.1371/journal.pone.0273651)

Multivariable logistic regression model

ChageGpe is a confounder for Deworming, modifying 15.07% of the crude OR
MothAge is a confounder for Deworming, modifying 10.41% of the crude OR

```
logistic anaemia chsex ib2.ChageGpe ib2.rezhill ib2.MothAge ib1.Deworming2 i.CgChrelat
Under5sibl i.CgEducGp ib3.SocEcoStat ib1.csfpfood Stunting Underweight i.malarscrn ,
vce(cluster rezhill)
```

```
Logistic regression                             Number of obs     =        456
                                                Wald chi2(2)      =          .
                                                Prob > chi2       =          .
Log pseudolikelihood = -236.11838               Pseudo R2         =     0.0944

                                  (Std. Err. adjusted for 4 clusters in rezhill)
```

anaemia	Odds Ratio	Robust Std. Err.	z	P>|z|	[95% Conf.	Interval]
chsex	.7217499	.1873934	-1.26	0.209	.4338932	1.200579
ChageGpe						
6-11	2.270164	.6298436	2.96	0.003	1.317943	3.910372
rezhill						
Budahunga	3.199367	.0697661	53.33	0.000	3.065509	3.339071
Mukenke	2.815694	.1655375	17.61	0.000	2.509241	3.159574
Mukenke II	2.189391	.2039741	8.41	0.000	1.823985	2.627999
MothAge						
<20	1.235	.3370486	0.77	0.439	.7233726	2.10849
>40	1.748757	.7670255	1.27	0.203	.740257	4.131203
Deworming2						
No	3.544047	1.227834	3.65	0.000	1.797219	6.988724
Not Applic	1	(omitted)				
CgChrelat						
Other relationship	2.705354	2.919235	0.92	0.356	.3263835	22.42437
Under5sibl	1.121374	.1045642	1.23	0.219	.9340689	1.346239
CgEducGp						
Secondary	.6710735	.1189211	-2.25	0.024	.474164	.9497552
tertiary	.4830618	.0592815	-5.93	0.000	.3797895	.614416
SocEcoStat						
Very poor	.8545814	.3872339	-0.35	0.729	.3516018	2.077092
Poor	.813839	.314508	-0.53	0.594	.3815855	1.735742
Rich	.8028312	.1891474	-0.93	0.351	.5059187	1.273995
csfpfood						
No	.4247728	.2260221	-1.61	0.108	.1497033	1.205264
Stunting	.8878957	.1917353	-0.55	0.582	.5815011	1.355731
Underweight	.7701928	.086612	-2.32	0.020	.6178423	.9601107
malarscrn						
Positive	2.487226	1.306162	1.74	0.083	.8886036	6.961812
_cons	6.250245	5.169144	2.22	0.027	1.23574	31.61308

estat classification, all

Logistic model for anaemia

Classified	True D	True ~D	Total
+	324	102	426
-	14	16	30
Total	338	118	456

Classified + if predicted Pr(D) >= .5
True D defined as anaemia != 0

Sensitivity	Pr(+| D)	95.86%
Specificity	Pr(-|~D)	13.56%
Positive predictive value	Pr(D| +)	76.06%
Negative predictive value	Pr(~D| -)	53.33%
False + rate for true ~D	Pr(+|~D)	86.44%
False - rate for true D	Pr(-| D)	4.14%
False + rate for classified +	Pr(~D| +)	23.94%
False - rate for classified -	Pr(D| -)	46.67%
Correctly classified		74.56%

Lroc

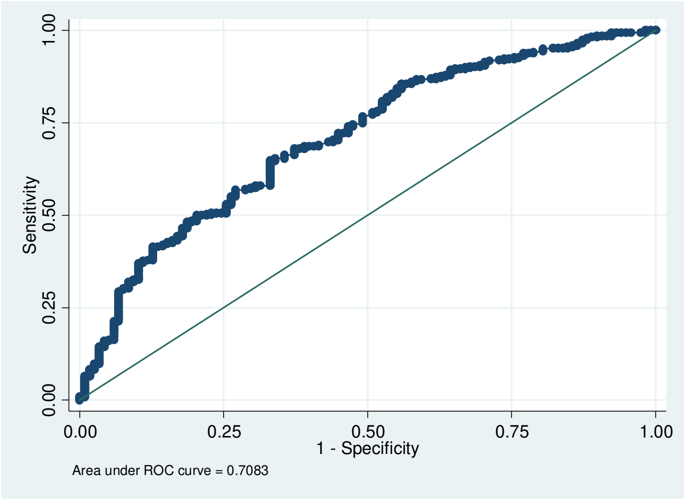

Supplement: S1 File — (PDF) [file pone.0273651.s001.pdf]
