# Supplementary material for: Prevalence and factors associated with anaemia in children aged 6–24 months living a high malaria transmission setting in Burundi
Source: PLoS One. 2022 Sep 2;17(9):e0273651. doi: 10.1371/journal.pone.0273651 (PMC9439186; doi:10.1371/journal.pone.0273651)
Supplement: S2 File — (PDF) [file pone.0273651.s002.pdf]

Effect of Micronutrient supplementation on anaemia and cognition among children in high malaria transmission setting in Burundi; a quasi-experimental study

Sub-study1 Questionnaire

Objective: To determine the prevalence and factors associated with anaemia in children aged 6-24 months and living in a high malaria transmission settings in Burundi

Instructions: *For child 6-24 months old whose caregiver has provided written informed consent to participate in the study. The key respondent should be the mother, if not available, then a primary caregiver (parent or adult who assumes the greatest responsibility in caring for the health and well-being of the child) should be the respondent.*

Note: If the caregiver has more than one eligible child in the household, randomly select one child (*by assigning number 1, 2 and 3 or higher depending on the number of children on sheets of paper. Fold the papers and ask their mother/caregiver to randomly select one*).

Date of interview (dd/mm/yyyy) : …………/………./……………

Health District : …………………………… Commune : ……………………………

Interviewer's ID: ………………. Supervisor's ID : ………………..

Section1: Household Information, Demographic and Socioeconomic Characteristics

Instructions: *Let us start with questions about demographic and socioeconomic characteristics of your household.*

Table 1.1: Household, Household head and caregiver information

	**Questions**	**Codes/Responses**	**Variable name**
1.1.1.	Household study ID	☐☐/☐☐/☐☐☐☐	HHSTUDI
1.1.2.	Hill/Colline	…………………………….	HCOLIN
1.1.3.	Village	…………………………….	HVILA
1.1.4.	Initials of Child	☐☐☐☐☐	CHINIT
1.1.5.	[1]Household head's initials	☐☐☐	HHDINIT
1.1.6.	Sex of the household head	1=male 2=female	HHSEX
1.1.7.	How old is the household head?<br>***Probe: How old was (name) at his / her last birthday? Record age in completed years***	Age (in completed years)<br>____ ____	HHDAGE
1.1.8.	Is the primary caregiver (respondent) also the household head?<br>***(skip to 1.1.14 if answer is "yes")***	1=yes, 0=no	HCGHEAD
1.1.9.	Marital status of household head	1=married or living together,<br>2= divorced/separated,<br>3= widowed<br>4= never married or never lived together	HHDMARTS
1.1.10.	Has the head of household been scholarly educated<br>*If **yes**, continue asking (1.1.11), If **no**, skip to 1.1.12*	1=yes, 0=no	HHDEDUC

1.1.11.	What is the education level of the Household head	(options below)<br>0 = None<br>1 = primary<br>2 = "O" level<br>3 = "A" level<br>5 = university/tertiary<br>99 = don't know	HHDEDU CLEV
1.1.12.	What is the occupation of the household head? That is, what kind of work does he mainly do?	1= no occupation,<br>2= agriculture/farming<br>3= sales and services<br>4= professional/technical/managerial<br>5=Handicraft and trade<br>6= plant/machine operator<br>7= house/domestic workers<br>9= Others (Specify)…………..	HHOCCU P
1.1.13.	Primary Caregiver's (respondent's) initials	□□□□□	HCGINIT
1.1.14.	How old is the caregiver?<br><br>*Probe: How old was (name) at his / her last birthday? Record age in completed years.*	Age (in completed years)<br>____ ____	HCGDAG E
1.1.15.	Sex of primary caregiver	1=male 2=female	HCAGSEX
1.1.16.	Primary caregiver relationship with the household head		HCGHHR EL
1.1.17.	Primary caregiver`s marital status	1=married, monogamous; 2=married, polygamous; 3=cohabiting; 4=single; 5=widowed; 6=divorced; 7=separated	CGMART S
1.1.18.	Primary caregiver relationship with the child	01 = Mother 02 = Father<br>03 = Grand-parent 04 = Brother/Sister<br>05 = Aunty or other relative<br>06 = adopted/foster/stepchild<br>07 = not related 99 = don't know	CGRELAT
1.1.19.	What is your religion? (Primary caregiver)	1=No religion<br>2= Christian (Catholic/Protestant/born again/evangelical)<br>3=Muslim 4=Jehovah's witness<br>5=Traditional<br>6=Other (specify) …………………	CGRELIG
1.1.20.	What is the education level of the primary caregiver	(options below)<br>0 = None 1 = primary<br>2 = "O" level 3 = "A" level<br>5 = university/tertiary 99 =Don't know	CGEDUCL EVEL
1.1.21.	What is your occupation? That is, what kind of work do you mainly do?	1= no occupation,<br>2= agriculture/farming<br>3= sales and services<br>4= professional/technical/managerial<br>5=Handicraft and trade<br>6= plant/machine operator<br>7= house/domestic workers<br>8= Others (Specify)…………..<br>99 =Don't know	CGOCCUP
1.1.22.	Parity (only for primary caregiver who are mothers) How many children do you have?	1= one child, 2=two children, 3= three children, 4= four children, 5= five and above	CGPARIT Y

1.1.23.	Is mother/caregiver pregnant or breast feeding?	1= Pregnant, 2= Breastfeeding (lactating) 3= Pregnant and breastfeeding, 4= None of the above	CGPREGBF
1.1.24.			
1.1.25.	Sex of Child	1=male 2=female	CHSEX
1.1.26.	How old is the child? (Date of birth)<br><br>Probe: *Record age in completed months (or date of birth).*	Age (in completed months) (date)<br>____ ____ …../…./….	CHAGE

[1]**Household head is defined as** the one who manages the income earned and expense incurred by the household, and is considered by other members of the household as the head**.** The household head could either be male or female, and is not necessarily the oldest person in the household.

[2]**A household** is defined as a group of people who routinely eat out of same pot and live on the same compound (or physical location). It is possible that they may live in different structures.

Table 1.2 Household Head/Caregiver Income

Instruction: Following are questions about income and the sources of income of this household.

No	Questions	Codes/Responses	Variable name
	Do you (Caregiver) earn any income?	1=yes 0=no	EARNINCOME
	During the past 30 days, what were your (caregiver) most important livelihood sources? (use income source codes, up to 3 activities)	**Most important** ______<br>**Second** *(leave blank if none)* ______<br>**Third** *(leave blank if none)* _______	INCOMESOURCES
	Does the household head earn any income?	1=yes 0=no	HHEARNINCOME
	During the past 30 days, what were most important livelihood sources of the household head? (use income source codes, up to 3 activities)	**Most important** ______<br>**Second** *(leave blank if none)* ______<br>**Third** *(leave blank if none)* _______<br>**Second** *(leave blank if none)* ______<br>**Third** *(leave blank if none)* _______	HHINCOMESOURCES

Income source codes:
1 = Food crop production/sales (e.g. maize)
2 = Cash crop production/sale (e.g. coffee)
3 = Income derived from sale of livestock and / or animal products
4 = Agricultural wage labor
5 = Non-agricultural wage labor (construction…)
6 = Sale of firewood/charcoal
7 = Petty trade (market, whackers, etc.)
8 = Pension, government allowances
9 = Salary
10 = Fishing / Hunting
11 = Handicrafts
12 = Gifts/begging
13 = Food assistance
14 = Brewing
15 = Remittances
16= Other

1.2.5	If answer to question is 15(Remittances), please indicate where the remittances were received from	1. Main town in the province<br>2. Neighbouring province<br>3. Other province/town within Burundi<br>4. Country outside Burundi<br>5. Other(specify):……….	REMITSOURCES

Table 1.3. Household members (including the respondent /caregiver)

Instructions: *These questions are regarding the home / the place where the child usually stays. Include all routine household members in the table below. Include household members who are temporarily away, working outside the home, or at boarding school, but exclude visitors who have stayed for less than 3 months.*

No	Questions	Codes/Responses	Variable name
	Total number of household members	…………………..	HHMEMBER
	Members of household who are under five year old	…………………..	HHU5MEMB

Table 1.4 Type of House

Instructions: *I am going to ask you questions about the type of house your children and you live in.*
(**Research Assistant**: *confirm responses with your observation.)*

No	Main house	Codes/Responses	Variable name
1.4.1.	Is the house	1= Owned 2=Rented<br>3=Other (specify________)<br>99=Don't Know	HZOWNSHIP
1.4.2	Main type of walls	1. Wood and mud 2.Mud bricks/burnt bricks<br>3.Concrete blocks 4.Wood<br>5.Grass/bamboo 6.Other (specify)……….	HWALL
1.4.3	Main type of roof	1. Grass thatched 2.Corrugated iron sheets<br>3. Tiles 4.Wood covered with mud/dung 5. Other (specify)………….	HROOF
1.4.4	Main type of floor	1.Mud or dirt 2. Brick/stones/cements<br>3.Tiles 4. Other (specify)………..	HFLOOR
1.4.5	What type of fuel does your household mainly use for cooking?	1=Wood 2=Straw/Shrubs/Grass<br>3= Charcoal 4= Gas or biogas<br>5= Electricity 6= Agricultural crop<br>7=Animal dung 9=Other(specify)………..	HHFUEL
1.4.6	Where do you cook from?<br>Do you have a separate room which is used as a kitchen?<br>**Or** *is the cooking usually done in the house, in a separate building/ outdoors?*	1= In the house 2= In another building<br>3= Outdoor 4=Other (specify)………..	HHCOOKPLC
1.4.7	What is your household`s main source of energy for lighting?	1.Electricity 2.Solar<br>3.Gas 4.Paraffin (lantern)<br>5.Paraffin (tadowa/koroboyi)<br>6.Open firewood place<br>7. Torch<br>8. Other (specify)…………………..	HHLIGHT
1.4.8	Does your household own any of the following assets?<br>1 – Yes, 0 – No<br><br>(*Enter '1' for yes, '0' for no*)<br>***WHILE ASKING, ALSO OBSERVE***	1.Electricity____<br>2.Radio/Tape___<br>3.Television____<br>4.Cell Phone___<br>5.A non-mobile telephone ____<br>6.Refrigerator ______	HHASSET
1.4.9	Does anyone in your household own any of the following assets?<br>1 – Yes, 0 – No<br><br>***(Enter '1' for yes, '0' for no)***	1.Bicycle ___<br>2. Animal-drawn cart ___<br>3. Motorcycle/scooter _____<br>4. Car/track ____<br>5.Boat with motor ______	HHMBASSET

1.4.10	Does your household own any of the following livestock?<br>***If 'no' skip to 1.4.12***	1=yes, 0=no		HHLIVESTOCK
1.4.11	If 'Yes', how many of the following livestock does your household currently own?	Livestock	Number	NOLIVESTOCK
		Cattle		
		Sheep		
		Goat		
		Pig		
		Chicken/Poultry		
		Other: (Specify)_____		
1.4.12	Does any member of this household own any agricultural land?	1=yes,<br>0=no		LANDOWNER
1.4.13	How many acres of agricultural land do members of this household own?	Acres ………………		LANDSIZE

Section 2: Water, Hygiene and Sanitation

Instructions: "*We are now going to touch on questions about how you get water and how you use it, how are organised household hygiene and sanitation*"

Table 2.1: Water, Hygiene and Sanitation

	**Questions**	**Codes/Responses**	**Var. name**
2.1	**Water use**		
2.1.1	What is the main source of drinking-water for members of your household?	1= Piped water through a tap<br>2= Water from open well/spring<br>3= Water from protected well/spring<br>4= Water from borehole fitted with a hand pump<br>5= Surface water (river, dam, run off, etc)<br>6= Rain water collected in a tank	WSOURCE
2.1.2	What is the main source of water used by your household for other purposes, such as cooking and hand washing?	1= Piped water through a tap<br>2= Water from open well/spring<br>3= Water from protected well/spring<br>4= Water from borehole fitted with a hand pump<br>5= Surface water (river, dam, run off, etc)<br>6= Rain water collected in a tank	WSOURCEUZ
2.1.3	Do you treat your water in any way to make it safer to drink?	☐ 1=yes<br>☐ 0=no	WTREAT
2.1.4	What do you usually do to the water to make it safer to drink? Anything else?	1=boiling,<br>2=use traditional herbs,<br>3=use chemicals (water guard, liquid),<br>4=filter/sieve,<br>5=decant,<br>6=other (specify) …………………………	TREATMTD
**2.2**	**Sanitation and hygiene**		

2.2.1	Does your household have toilet facilities?<br>*If "**no**", skip to 2.2.4*	☐ 1=yes<br>☐ 0=No	HHTOILET
2.2.2	What kind of toilet facilities do you use or, rather, have within the household and use?	1= Flush toilet<br>2= Pit Latrine with slab/VIP<br>3= Open pit (no super structure)<br>4= bucket latrine	TOILETYP
2.2.3	Do you share this toilet facility with other households?<br>*If yes, continue asking (2.2.4), If no, skip to 2.2.5*	☐ 1=yes<br>☐ 0=No	HSHARTOILT
2.2.4	How many households, including yours, use this toilet facility?	☐1= Not shared<br>☐2= Two HH<br>☐3= 3 HH or more<br>☐9 = Don't know	TOILETSHAR
2.2.5	Under what circumstances do you wash your hands? (***Do not read the responses below. Allow respondent to answer, then fill in 1=yes for the last answer (appropriate) and 0=No for other responses***)		
	Not at all	1	WHNDWASH
	When they have visible dirt	2	
	After toilet use/defecation/urination	3	
	After cleaning child following defecation	4	
	Before preparing the food	5	
	Before serving a meal	6	
	Before eating	7	
	Before feeding a child	8	
	When I am reminded to do so	9	
	Before/after caring for someone who is sick	10	
	(1) Before, during, and after preparing food (2)After toilet/After cleaning child following defecation, (3) Before eating or feeding a child, (4) Before and after caring for someone who is sick	11	
2.2.6	What do you usually use to wash your hands in your household?	0=Water 1=Water and soap<br>2=water & ashes 3=Other (specify)	WTHANDWASH
2.3	**Child stool disposal**		
2.3.1	The last time (name) passed stools, what was done to dispose of the stools?	1= Child used toilet / latrine 01<br>2= Put / Rinsed into toilet or latrine 02<br>3= Put / Rinsed into drain or ditch 03<br>4= Thrown into garbage (solid waste) 04<br>5= Buried 05<br>6= Left in the open 06<br>7= Other (*specify*)………………….. 07<br>99= Don't know 99	STOOLDISPOSAL

CHILD FEEDING, HEALTH AND MORBIDITY AND ANTHROPOMETRY

Instruction: What about feeding, health and illnesses of your child. I will take body measurement of your child.

Section 3: Child Feeding *(to be administered to mother / care giver of the child)*

Table 3.1: Breastfeeding and complementary

	**Question**	**Responses**	**Var. name**
3.1.1	Has this (NAME) ever been breastfed?	☐ 1=Yes ☐ 0=No	CFBREAST
3.1.2	If yes how long after birth (NAME) first put on breast?<br>***If less than 1 hour, record '00' hours.***<br>***If less than 24 hours, record hours.***<br>***Otherwise, record days***	Immediately __ ___00<br>Hours ___ ___<br>Days ____ ___	CFBFEDBIRTH
3.1.3	In the first seven days after delivery, was (NAME) given anything to drink other than breast milk?	1= yes, 0= no	CFRSEVEN
3.1.4	What was (NAME) given to drink?<br>Anything else? (circle all drinks mentioned)	1=Milk (other than breast milk)<br>2=Plain water<br>3=Sugar or glucose water<br>4=Sugar-salt-water<br>5=solution<br>6=Fruit juice<br>7=Infant formula<br>8=Tea/infusions<br>9=Coffee<br>10=Honey<br>11=Local brew<br>12=Other (specify) …………….	CFRTHREDRK
3.1.5	Is (NAME) currently breastfeeding?<br>*(if answer "yes" skip to 3.1.6, if it is "no" continue to 3.1.6)*	☐ 1=yes ☐ 0=No	CFBNOW
3.1.6	How old was (NAME) when (NAME) completely stopped breastfeeding or being fed breast milk?	___months	CFAGE
3.1.7	Reason why did the mother stop breastfeeding?	1= Mother pregnant<br>2= Mother sick<br>3= Mother tired of breast feeding<br>4= Introduced solids<br>5= Breast milk making child sick<br>6= Not enough breast milk<br>7= Other Please specify_______ __	CFBRZNSTOP
3.1.8	How many times did you breast feed (NAME) yesterday (from time you woke up this morning till night?	________times	CFTIMES
(This next question is about the first thing that [child] was given other than breast milk: complementary feeding/formula).			

3.1.9	Did (NAME) was given other than breast milk/ Did (NAME) started complementary feeding	☐ 1=Yes<br>☐ 0=No	CFEDOTHER
3.1.10	How old was (NAME) when (he/she) started complementary feeding	______ months	CFEDFORMULA

Table: 3.2: Food Groups

No	Questions	Codes/Responses	Var. names
	Which foods did you give to (NAME) since yesterday, during day and at night? *(respond to all below)*	☐ 1=Yes ☐ 0=No<br>☐ 9=DK	
3.2.1	Rice, bread, cake and/or donuts, sorghum, millet, maize or porridge of rice, sorghum, millet, maize		FCEREAL
3.2.2	Pumpkin, carrots, squash, or sweet potatoes that are yellow or orange inside		FVITA
3.2.3	White potatoes, white yams, manioc, cassava, or any other foods made from roots		FROOTS
3.2.4	Any dark green leafy vegetables		FGREENS
3.2.5	Ripe mangoes, papayas, bananas or Japanese plums, maracuja, pineapple, oranges, avocados, strawberries		FFRUITS
3.2.6	Any other fruits or vegetables		FVEGS
3.2.7	Liver, kidney, heart, or other organ meats		FORGANMT
3.2.8	Any meat, such as beef, pork, lamb, goat, chicken, or duck		FMEAT
3.2.9	Eggs		FEGG
3.2.10	Fresh or dried fish or shellfish/crustacean		FFISH
3.2.11	Any foods made from beans, peas, lentils, nuts, or seeds		FPULSE
3.2.12	Cheese, yogurt, or other milk products		FMILK
3.2.13	Any oil, fats, or butter, or foods made with any of these		FOIL
3.2.14	Any sugary foods such as, sweets, candies, pastries, cakes, or biscuits		FSUGAR
3.2.15	Grasshoppers, ants, termites, crabs, snails, or other insects		FINSECTS
3.2.16	Any other (specify) …………………..		FANYOTHER
**3.3. Other Questions**			
3.3.1	Yesterday, during the day or night, did (NAME) consume any fortified or rehabilitation products (CSB, Plumpy'nut, Fortified flour from UN Agency or NGO distribution)?	1= yes, 0=no	CSFPFOOD

3.3.2	Who prepares/feeds (NAME)?	1=primary caregiver/mother<br>2=grandmother<br>3=father<br>4= Sibling<br>5=child<br>6= others (specify)………..	CHDPREFEED
3.3.3	How many hours a day caregiver spends with (NAME)?	_____hours	CTIMESPENDS

Section 4: Child Health Status and Morbidity

Ask the caregiver if she can share the child's health card. Ask the following questions about the ***<u>child.</u>***

Table 4.1: Child Health Status

	Question	Responses	Var. name
4.1.1	Do you have a book where (NAME)'s vaccinations are written down?	☐ 1=Yes<br>☐ 0=No	CVACBOOK
4.1.2	Has (NAME) ever received a BCG vaccination against tuberculosis – that is, an injection in the arm or shoulder that usually causes a scar?	1= Yes, with book<br>2= Yes, without book<br>3= No, with book<br>4=No, without book<br>99=Do not know	CBCG
4.1.3	Has (NAME) ever received any "vaccination drops in the mouth" to protect him/her from getting diseases – that is, polio?	☐ 1=Yes ☐ 0=No<br>☐ 99=DK	CPOLIO
4.1.4	How many times was the polio vaccine received?	Number of times……..	CNOAGPOLIO
4.1.5	Has (NAME) ever received a Pentavalent vaccination – that is, an injection in the thigh or buttocks – to prevent him/her from getting tetanus, whooping cough, diphtheria, *Haemophilus influenzae* type b and Hépatitis B?<br><br>*Probe by indicating that DPT vaccination is sometimes given at the same time as Polio.*	☐ 1=Yes<br>☐ 0=No<br>☐ 99=DK	CDPENTAV
4.1.6	How many times was a Pentavalent vaccine received?	Number of times……	CNOPENTAV
4.1.7	Has (NAME) ever received a Measles injection or an MMR injection – that is, a shot in the arm at the age of 9 months or older and 18 Months - to prevent him/her from getting measles?	☐ 1=Yes<br>☐ 0=No<br>☐ 99=DK	CMEASLES
4.1.8	How many times was a DPT vaccine received?	Number of times……..	CMEASLESTIME

4.1.9	Has (NAME) ever been given a Rotavirus vaccination – that is, liquid in the mouth– to prevent him/her from getting diarrhoea?	☐ 1=Yes<br>☐ 0=No<br>☐ 99=DK	CROTAVIRUS
4.1.10	How many times was a ROTAVIRUS vaccine received?	Number of times……..	CROTATIME
4.1.11	Has (NAME) ever been given a Rotavirus vaccination – that is, an injection in the thigh or buttocks – to prevent him/her from getting pneumonia?	☐ 1=Yes<br>☐ 0=No<br>☐ 99=DK	CPCV
4.1.12	How many times was a PCV vaccine received?	Number of times___________	CPCVTIME
4.1.13	Has the (NAME) received a vitamin A capsule in the last six months?	☐ 1=Yes ☐ 0=No<br>☐ 99=DK	CVITA
4.1.14	Has the (NAME) been de-wormed in the last six months?	☐ 1=Yes ☐ 0=No<br>☐ 99=DK	CDEWORM
4.1.15	What is the birth order of (NAME)?	1= first, 2= second, 3= third, 4=fourth, 5= fifth, 6=sixth & above	CBIRTHORDER
4.1.16	When (NAME) was born, was he/she very large, larger than average, average, smaller than average, or very small?	1=very large,2=larger than average, 3=average, 4=smaller than average, 5=very small, 99=Don't know	CBIRTHWGT
4.1.17	Was (NAME) weighed at birth?<br>If yes, go to 6.1.8.	☐ 1=Yes ☐ 0=No<br>☐ 99=DK	CWEIGHED
4.1.18	How much did (NAME) weigh?	Record weight in Kg from Health Card, if available<br><br>1. Kg from card……..<br>2. Kg from recall…….	CWEIGHT
4.1.19	Copy dates from the card. Write ' 44' in 'day' column if card shows that a dose was given, but no date is recorded.		

	Day		Month		Year				Expected Time	CIMMCOMPL
BCG									Birth	
Polio 0										
Polio 1									6wks	
DPT-HebB+Hib1 (Pentavalent 1)										
Pneumococcal conjugate vaccine (**PCV13**) 1										
Polio 2									10 wks	
DPT-HebB+Hib2 (Pentavalent 2)										
Pneumococcal conjugate vaccine (**PCV13**) 2										
Polio 3									14 wks	
DPT-HebB+Hib3(Pentavalent 3)										
Pneumococcal conjugate vaccine (**PCV13**) 3										
Rotavirus 1									6 months	
Measles 1									9months	
Rotavirus 2									10months	

	Measles 2									18months	
	Vitamin A (Most recent)										

Table 4.2: Child Morbidity

Instructions: *"We are going to talk about how illnesses and symptoms your child1 had in the last two weeks"*

**No**	**Question**	**Codes/Responses**	**Var. name**
4.2.1.	Has the (NAME) had any illness in the past two weeks? ***If the answer to 5.2.2 is "Yes", then continue below. Otherwise, skip to the next section.***	☐ 1=Yes<br>☐ 0=No	CILL
4.2.2	Which illnesses or symptoms has (NAME) had in the past two weeks? ***Do not read the list below; allow the caregiver to answer then complete all questions in the table.***	For how many days in the past two weeks (14 days), has the child experienced the following:	
4.2.3	Cough	☐☐ days	CCOUGH
4.2.4	Difficult or fast breathing	☐☐ days	CFASTBEARTH
4.2.5	Fever	☐☐ days	CFEVER
4.2.6	Diarrhoea without blood	☐☐ days	CDIARRH
4.2.7	Diarrhoea with blood	☐☐ days	CBLOODIARRH
4.2.8	Other (specify …………………..)	☐☐ days	CILLOTHER

Table 4.3: Treatment-seeking practices and case management

	**Question**	**Responses**	**Var. name**
4.3.1.	If (NAME) had any illness at any time, what is your treatment-seeking choice?<br><br>*Where do you take him for his treatment?*<br>***If the answer to 4.3.1 is "1" or "2", continue below. Otherwise, skip to 4.4 section.***	1= Health facility (public, private or religious conventional)<br>2= Community Health Workers (iCCM)<br>3= traditional healer or witchdoctor<br>4= prayer room<br>99=Do not know	TTSEEKING
For the specific illnesses or symptoms has (NAME) had in the past two weeks, what was the treatment did they gave you?		For the specific illnesses or symptoms has the child, is the following the (According to national guidelines standard/treatment) :	
4.3.2	Cough	Treatment…………………	TTCOUGH
4.3.3	Difficult or fast breathing	Treatment…………………	TTBREATH
4.3.4	Fever	Treatment…………………	TTFEVER
4.3.5	Diarrhoea without blood	Treatment…………………	TTBDIARRH
4.3.6	Diarrhoea with blood	Treatment…………………	TTBLOODIARRH
4.3.7	Have you ever lost a child 6-24 months old	☐ 1=Yes ☐ 0=No	CHILDLOST

4.4. Health facility access and Community Health Worker support

	Question	Responses	Var. name
4.4.1	If you need to go to the nearest health facility, which transportation do you use?	1= Privet car/motorcycle<br>2= Public (Taxi, bus, motorcycle)<br>3= Foot 4= Bicycle<br>5= Other (specify) ……………..	HFACCES
4.4.2	How long does it take to get to the nearest health facility (using transportation mentioned in the question above)	1= 0-30 minutes 2= 31-60 minutes<br>3= 61-120 minutes 4=more than 120min<br>99= Don't know	HFTIME
4.4.3	Do you have Community Health Workers-CHW (or light mothers) providing you nutritional education in your village?	☐ 1=Yes<br>☐ 0=No	CHWNUT

Section 5: Anaemia, malaria and helminthes diagnosis/testing

Table 5.1: Anaemia

Instructions: *"Now, we are going to discuss about symptoms and histories about anaemia and malaria that your child1 may had in the last two weeks. I am also going to take two drops of blood from a finger prick (or a heel prick in the case of children age 6-11 months) to analyse haemoglobin and screen for malaria. There are no serious risks associated with the procedures, your child may feel minimal pain"*

5.1	**Haemoglobin testing**	**Results**	**Var. name**
5.1.1	Did (NAME) suffered from anaemia last 2 weeks?	☐ 1=Yes ☐ 0=No<br>☐ 99=DK	ANAEMIAHST
5.1.2	Conjunctival and/or palmar pallor (Clinical exam)	☐ 1=Yes<br>☐ 0=No	ANAEMIAPALLOR
5.1.3	Haemoglobin testing (using HemoCue analyser)	**……. g/l**	HBSTATUS

Table 5.2 Malaria

5.2.1	Did (NAME) got any malaria episode last six months<br>*Do not read the definition below*; ask the caregiver to define which diagnosis and then complete the number of episodes*<br>***If the answer to 5.2.1 is "Yes", continue below. Otherwise, skip to 5.2.5.***	☐ 1=Yes<br>☐ 0=No	MALAREPIZD
5.2.2	Which parasitological method of diagnosis did they used	☐1= blood smear<br>☐2= Rapid diagnosis test (RDT)<br>☐8= Do not know	MALARSCRN
5.2.3	What is the amount of malaria episode did (NAME) got	☐1= One episode ☐2= 2 to 3<br>☐3= more than 3 ☐99=Don't remember	MALAREPIZNO
5.2.4	Which treatment did (NAME) got at the last episode of malaria	☐1=Artemisinin-based combination therapy<br>☐2= Quinine<br>☐3= Other, Specify (…………………)<br>☐99=Do not remember	MALAREPIZTT

5.2.5	**Fever** (Axillary or 24 hours history): *Did (NAME) suffered from fever last 24 hours?* Or *axillary temperature*	☐ 1=Yes<br>☐ 0=No<br>…… **°C**	FEVER
5.2.6	Malaria screening (using RDT)<br>***Prick a finger (or a heel prick in the case of children age 6-11 months) and use blood drops to make the RDT according to Lab POS***	☐ 1=Positive<br>☐ 0=Negative	MALARSCRN
5.3	**Helminthes testing**	**Results**	**Var. name**
5.3.1	Helminthes testing (using microscopic detection by wet preparation and Kato-Katz technique)	☐ 1=Positive<br>☐ 0=Negative<br>**……. g/l**	HELMTEST
5.3.2	If helminthe test is positive, which parasite has been identified	…………………………	PARASITYPE

***Malaria episode**: a history of fever in the last 24 hours or documented axillary temperature $\geq$ 37.5°C in presence of a positive malaria blood test*

Section 6. Anthropometric measurements

Instructions: *The children will be measured two times and records of measurements will be taken until consistent results obtained across assistants at least one unity point (MUAC, height/length) or decimal point (Weight).*

No	Measurement	Results	Var. name
6.1.1	Bilateral Pitting Oedema Screening	☐ 1=Present<br>☐ 0=Absent<br>☐ 8=not sure	**CBPOSCREEN**
6.1.2	**MUAC (cm)**	Interviewer 1 ……….cm ………cm<br>Interviewer 2 ……….cm ………cm	MUAC
6.1.3	**Weight (Kg)**	Interviewer 1 ……….Kg ………Kg<br>Interviewer 2 ……….Kg ………Kg	WEIGHT
6.1.4	**Length/Height (cm)**<br>Instructions: *Measure children younger than age 24 months in lying down position on the board (recumbent length), and measure older children in standing position.*	Interviewer 1 ……….cm ………cm<br>Interviewer 2 ……….cm ………cm	LENGTH
		Interviewer 1 ……….cm ………cm<br>Interviewer 2 ……….cm ………cm	HEIGHT

THANK YOU FOR YOUR VALUABLE TIME

	Interv/Examiner 1	Interv/Examiner 2	Supervisor/Quality control agent	Data entry #1	Data entry #2
Code/Initials	|__|__|/______	|__|__|/______	|__|__|/_______	|__|__|/______	|__|__|/_______
Date	|__|__|/|__|__|/20|__|__|	|__|__|/|__|__|/20|__|__|	|__|__|/|__|__|/20|__|__|	|__|__|/|__|__|/20|__|__|	|__|__|/|__|__|/20|__|__|
