# Supplementary material for: Prevalence and factors associated with anaemia in children aged 6–24 months living a high malaria transmission setting in Burundi
Source: PLoS One. 2022 Sep 2;17(9):e0273651. doi: 10.1371/journal.pone.0273651 (PMC9439186; doi:10.1371/journal.pone.0273651)
Supplement: S3 File — (PDF) [file pone.0273651.s003.pdf]

Akamaro ko kwongereza ingaburo mu mfungurwa ziherezwa i muhira hakoreshejwe « INGABURO IBEREYE » kw'igabanuka ry'amaraso n'ubushobozi bwo kwiga, gutahura no gusobanura mu bana bafise amezi atandatu n'abazokwiza imyaka ibiri ejo mu turere n'imihingo birwiriyemwo malariya kurusha ahandi mu Burundi : icirwa c'ubushakashatsi

Igice cambere c'icirwa

Intumbero: Kwerekana uko kubijanye n'uko ibitigiri vv'abana barwaye igabanuka ry'akamaro n'ivyoba bituma iryogabanuka ryiyongera mu turere n'imihingo birwiriyemwo malariya kurusha ahandi mu Burundi

Uko bitunganijwe: Abana bafise amezi atandatu n'abazokwiza imyaka ibiri ejo, abavyeyi babo canke abarezi babo biyemeje kuja muri kino cirwa nibo baheza bakaduha ikigniro. Uwishura ibibazo ni nyina w'umana canke umurezi (umuntu akuze) ajejwe kubungabunga umwana canke asanzwe yitaho nya mwana mu buzima bwiwe bwa misi yose.

Menya neza: Mu gihe urugo rurimwo abana babiri bashobora kuja muri kino cirwa, n'ugutoramwo umwe gusa hakoreshejwe itombora/igihamana. *Kubaha inomero 1 na 2, kuzandika ku dupapuro, ukatuzinga hanyuma ugasaba ko nyina w'abo bana canke uwujejwe kubabungabunga atoramwo kamwe gusa. Inumero itorwamwo niwe mwana azoheza akaja muri kino cirwa.*

Umusi w'ikiganiro : …………/………./……… Akarere k'ubuvuzi : ……………………………

Komine : ……………………………

Umwidondoro w'uwakoresheje ikiganiro : ……… Umwidondoro w'umugenduzi: …………………..

Ikigabane ca: Ibijanye imibereho n'ibiranga urugo rutuyemwo umwana

Instructions: Nashaka munkundire duhere ku bijanye imibereho n'ibiranga urugo umwana atuyemwo.

Table 1.1: Ibijanye n'urugo ha mwe n'umukuru warwo (uwurongoye uru rugo)

	Ibibazo	Inyishu zatanzwe/zishoboka	Inyishu
1.1.1.	Umwidondoro w'urugo	□□/□□/□□□	HHSTUDI
1.1.2.	Umutumba	…………………………….	HCOLIN
1.1.3.	Agacimbiri	…………………………….	HVILA
1.1.4.	Indome zitangura amazina y'umwana	□□□□□□	CHILDID
1.1.5.	[1]Indome zambere zigize izina n'amatazirano vy'uwurongoye urugo	□□□	HHDINIT
1.1.6.	Igitsina c'uwurongoye urugo	1=Gabo 2=Gore	HHDSEX
1.1.7.	Imyaka y'uwurongoye urugo<br>***Uwurongoye urugo afise imyaka ingahe?***<br>***Andika imyaka amaze gukwiza.***	Imyaka (amaze gukwiza)<br>____ ____	HHDAGE
1.1.8.	Uwurongoye urugo ni nawe asanzwe arera uyu mwana akamwitaho imisi yose?<br>***(inyishu ari «Ego », bandaniriza ku kibazo 1.1.13)***	|__|1=Ego, |__| 0=Oya	HCGHEAD
1.1.9.	Uwurongoye urugo arubatse ?	1=arubatse biciye mu mategeko canke abana n'umufasha<br>2= yarahukanye/yaravanye n'umufasha<br>3= umupfakare/kazi<br>4= Umusore/inkumi	HHDMARST

1.1.10.	Uwurongoye urugo yaraciye ku ntebe y'ishure ?<br>***Inyishu ari «Ego » bandanya ikibazo gikurikira 1.1.11***	|__|1=Ego, |__| 0=Oya	HHDEDUC
1.1.11.	Yagarukiye mu wakangahe canke afise uruhe rupapuro rw'umutsindo (iyihe mpamyabushobozi) ?	(imwe mu nyishu zikurikira)__________<br>1 = ntiyarangije amashure mato<br>2 = Amashure mato (6^{e})<br>3 = Igice ca 1c'amashure yisumbuye/ ECOFO<br>4 = Igice ca 2 c'amashure yisumbuye<br>5 = Kaminuza<br>99= ntavyo nzi	HHDEDUCLEV
1.1.12.	Uwurongoye urugo akora iki/uwuhe mwuga	1= nta kazi afise/nta mwuga akora<br>2= umurimyi/umworozi<br>3= umudandaza<br>4= umukozi wa leta/technicien/Umurwizatunga<br>5=Umunyamwuga 6= akazi ko mu nzu<br>7= Akandi kazi (vuga akariko)………….<br>99= Ntavyo nzi	HHOCCUP
1.1.13.	Indome zambere zigize izina n'amatazirano vy'uwujejwe kubungabunga umwana	□□□□□	HCGINIT
1.1.14.	Uwujejwe kubungabunga umwana afise imyaka ingahe ?<br>***Wewe ubungabunga umwana ufise imyaka ingahe?***<br>***Andika imyaka akwije***	Imyaka (amaze gukwiza)<br>____ ____	HCGDAGE
1.1.15.	Igitsina c'uwubungabunga umwana	1=Gabo 2=Gore	HCGSEX
1.1.16.	Ufitaniye (uwubungabunga umwana) ubuhe bucuti (iyihe migenderanire) n'uwurongoye urugo ?	01 = Umutambukanyi/umunega<br>02 = Umwana/umwishwa/umusengezana<br>03 = Umukwe/umukazana<br>04 = Umwuzukuru 05 = Umuvyeyi<br>06 = Sebukwe/inabukwe<br>07 = Murumunawe/mushikiwe<br>08 = Ubundi bucuti (inarume/senge/ umuvyara)<br>09 = Umurerano 10 = Nta bucuti bafitaniye<br>99 = Ttavyo nzi	HCGHHREL
1.1.17.	Urubatse (uwubungabunga umwana) canke nturubaka	1 = nubakanye n'uwufise umugore 1 ; 2 = ndi umugore agira …/mfise abagore … (polygamie); 3 = turibanira ntiturandikwa; 4 = Sindubaka célibataire; 5 = narapfakaye; 6 = narahukanye; 7 = twaratandukanye	CGMARST
1.1.18.	Ufitaniye (uwubungabunga umwana) ubuhe bucuti n'umwana ?	01 = sekuru/inakuru 02 = umuvyeyi (nyina/se)<br>03 = mukuru wiwe 04 = inasenge (inarume)<br>05 = nuwo tureze/ ndi mukase<br>06 = nta bucuti dufitaniye 99 = nta vyo nzi	CGRELAT
1.1.19.	Uri umuyoboke/usengera (uwubungabunga umwana) mu rihe dini?	1 = nta dini nyoboka<br>2 = Umukristu/umukristo (katolika/protestanti)<br>3 = Umu islamu 4 = Icabona ca Yehova<br>5 = Kiranga 6 = Irindi dini (vuga iryariryo)……	CGRELIG
1.1.20.	Waraciye (uwubungabunga umwana) ku ntebe y'ishure<br>***Inyishu ari «Ego » bandanya ikibazo gikurikira 1.1.20***	|__|1=Ego, |__| 0=Oya	CGEDUC

1.1.21.	Wagarukiye (uwubungabunga umwana) mu wakangahe/ufise uruhe rupapuro rw'umutsindo (iyihe mpamyabushobozi)	(imwe mu nyishu zikurikira)__________<br>1 = ntiyarangije amashure mato<br>2 = Amashure mato (6^e)<br>3 = Igice ca 1c'amashure yisumbuye/ ECOFO<br>4 = Igice ca 2 c'amashure yisumbuye<br>5 = Kaminuza 99= ntavyo nzi	CGEDUCLEVEL
1.1.22.	Ukora iki/uwuhe mwuga ?	1= nta kazi mfise/udukorwa tw'imuhira<br>2= umurimyi/umworozi 3= umudandaza<br>4= umukozi wa leta/technicien/manager<br>5=Umunyamwuga 6= akazi ko mu nzu<br>7= Akandi kazi (vuga akariko)………….<br>99= Ntavyo nzi	CGOCCUP
1.1.23.	Imvyaro (***ku babungabunga abana b'abakenyezi***)<br>Wibarutse abana bangahe?	1= Umwana umwe, 2=abana babiri, 3= abana batatu, 4= abana bane, 5= abana batanu n'abarenga	CGPARITY
1.1.24.	Usanzwe wibungenze canke wonsa ?	1= Ndibungenze, 2= Ndonsa 3= Ndibungenze kandi ndonsa 4=sinibungenze/sinonsa	CGPREGBF
1.1.25.	Igitsina c'umwana	1=Umuhungu 2=Umukobwa	CHSEX
1.1.26.	Umwana afise amezi angahe? (yavutse ryari)<br>***Andika amezi amaze gukwiza (canke itariki y'amavuka).***	Amezi (amaze gukwiza) (yavutse ryari)<br>____ ____ …../…./….	CHAGE

[1]**Uwurongoye urugo** n'uwusanzwe atunganya umutungo n'ibikoreshwa mu rugo vyose, yemerwa n'ababa murugo rugo nk'umukuru warwo. Ashobora kuba umugore canke umugabo, si ngombwa ngw'abe akuze gusumba abarubamwo bose.

[2]**Urugo** n'umurwi w'abantu basangira imfungurwa bakongera bakaba mu nyubakwa imwe canke zitandukanye ariko mu kigo kimwe.

Table 1.2 Umutungo w'urugo/w'uwubungabunga umwana

Instruction: Ibibazo bikurikira bijanye n'umutungo w'urugo/w'uwubungabunga umwana.

	**Ibibazo**	**Inyishu zatanzwe/zishoboka**	**Inyishu kabanga**
	Wewe ubungabunga umwana, Usanzwe ukorera amahera/ urahembwa?	|__|1=Ego, |__| 0=Oya	EARNINCOME
	Mu mezi atandatu aheze, uburyo/umutungo mwakoresheje (uwubungabunga umwana) wakomotse hehe ? (koresha inyishu, gushika kuri 3, zatanzwe aho hepfo)	**Inkomoko nyamukuru** ______<br>**Iyigira kabiri** *(atayihari siga ikibanza kigaragara)* __<br>**Iyigira gatatu** *(atayihari siga ikibanza kigaragara)* ___	INCOMESOURCES
	Uwurongoye urugo akorera umushahara/arahembwa?	1=Oui 0=non	HHEARNINCOME
	Mu mezi atandatu aheze, uburyo/umutungo uwurongoye urugo yakoresheje wakomotse hehe ? (koresha inyishu, gushika kuri 3, zatanzwe aho hepfo)	**Inkomoko nyamukuru** ______<br>**Iyigira kabiri** *(atayihari siga ikibanza kigaragara)* __<br>**Iyigira gatatu** *(atayihari siga ikibanza kigaragara)* ___	HHINCOME SOURCES

Inkomoko y'uburyo/umutungo:<br>*1 = igurishwa/umwimbu imbumbarugo (ak :ibigori)*<br>*2 = igurishwa/umwimbu njabukamazi (ak :ikawa)*<br>*3 = igurishwa ry'ibitungwa/umwimbu ukomoka mu bworozi*<br>*4 = kurimira ingero*	*5 = guca ingero mu bindi bikorwa atari uburimyi (ak :kwubaka…)*<br>*6 = igurishwa ry'inkwi/amakara*<br>*7 = urudandazwa ruto*<br>*8 = pensiyo, imfashanyo ya leta*<br>*9 = Umushahara (leta/ishirahamwe)*	*10 = Uburovyi*<br>*11 = umwuga*<br>*12 = ingabire/gusegereza*<br>*13 = imfashanyo y'ibifungurwa*<br>*14 = guhingura ibinyobwa*<br>*15 = irungikwa ry'amahera*<br>*16 = ubundi (vuga ubwaribwo)*

1.2.5	Inyishu ari 15 (irungikwa ry'amahera), ongerako iyo ayo mahera yaje azananye	1. Umurwa mukuru w'itara<br>2. Intara duhana imbibe<br>3. Iyindi ntara/igisagara co mu Burundi<br>4. Ikindi gihugu<br>5. Ahandi (vuga ahariho):……….	REMITSOURCES

Table 1.3. Ababa mu rugo (n'uwubungabunga umwana arimwo)

Instructions: *Ibibazo bikurikira bijanye n'abasanzwe babana n'umwana mu nzu imwe. Uzuza ababa mu rugo bose, ushiremwo abatahari, bakorera kure y'aho baba, abiga baba mu ndaro ariko ntushiremwo abamaze amezi arenga atatu (3) atariho baba.*

Uzuza igitigiri ukurikije igitsina n'imyaka.

1.3.1 Imyaka	Bose hamwe	Igitigiri c'igitsina gore	Encodage (HAGEGP)
Musi y'imyaka 2 (≥2)			HAGEGP1
Kuva ku myaka 2 kubatarakwiza 5 (2-<5)			HAGEGP2
Kuva ku myaka 5 gushika kuri 14			HAGEGP3
Hagati y'imyaka 15 na 17			HAGEGP4
Hagati y'imyaka 18 na 49			HAGEGP5
Hagati y'imyaka 50 na 64			HAGEGP6
Imyaka 65 n'iyirenga			HAGEGP7
**Bose hamwe**			**HHMEMBR**

Table 1.4 Uburaro

Instructions: Ngira ndababaze utubazo tujanye n'uburaro *(mugihe uriko uramubaza utubazo, raba neza kw'inyishu yaguhaye zijanye n'ivyo wibonera).*

No	Uburaro nyamukuru	Inyishu zatanzwe/zishoboka	Inyishu kabanga
1.4.1.	Iyi nzu mubamwo ni …<br><br>(***inzu mubamwo/mutuyemwo ni rwanyu***)	1= rwanyu<br>2=iyo mukodesheje (iyo muriha ku kwezi)<br>3=ibindi (vuga ivyarivyo________)<br>99=Sinzi ivyayo	HZOWNSHIP
1.4.2	Impome zayo zubatswe	1=ibiti/imbingo n'ivyondo<br>2=Amatafari mabisi/amatafari aturiye<br>3=Amaboroko sima (Blocs de béton)<br>4=ibiti 5=ivyatsi/imbingo/amarenga<br>6=Ibindi (vuga ivyarivyo) ……………….	HWALL
1.4.3	Iyi nzu mubamwo isakajwe	1. Ubwatsi/ibihunda/ibitokatoke 2. Amabati<br>3. Amategura 4. Ibiti hariko ivyondo/amase<br>5. Ibindi (vuga ivyarivyo)……………….	HROOF
1.4.4	Hasi mw'yi nzu mubamwo hari iki ?	1= Ivu isanzwe (isi y'umusenyi)<br>2= amatafari/amabuye/isima 3= Amategura<br>4=Ibindi (vuga ivyarivyo)…………………	HFLOOR
1.4.5	Mukoresha iki/mucana iki muguteka ivyo mufungura	1 = inkwi (ibiti) 2 = ibihunda /ivyatsi<br>3 = amakara 4 = gaze/biogaze<br>5 = umuyagankuba/amashanyarazi<br>6 = ibisigarira vy'ibiterwa 7 = amase	HHFUEL

		9=Ibindi (vuga ivyarivyo)…………………	
1.4.6	Murafise igikoni mukinjikiramwo? canke «***mukinjikira mu nzu canke mu gikoni***»	1= mu nzu 2= mu gikoni (cubatswe ukwaco)<br>3= hanze 4= Ahandi (vuga ahariho) …………..	HHCOOKPLC
1.4.7	Mukoresha iki mukubonesha/kumurika mu nzu iyo bwije ?	1. umuyagankuba<br>2. Amashanyarazi yimishwarara y'izuba<br>3. gaze<br>4. Itara ryikiyo rya peterori<br>5. agatadowa / agakoroboyi)<br>6. aho dukinjikira/inkwi/ikimuri 7=Itoroshi<br>8. Ikindi (vuga icarico) ……………… ..	HHLIGHT
1.4.8	Aha mu rugo iwanyu murafise igikoresho gikurikira?<br>1 – Ego/kirahari<br>0 – Oya/ntaco dufise<br>(*andika «**1**» kihari, «**0**» ata kiriho*)<br>***Ihweze neza kw'ivyo yemeje bisanzwe bihari***	1. Umuyagankuba ____<br>2. Iradiyo____<br>3. Imboneshakure/Terevisiyo____<br>4. Iterefone ngendanwa____<br>5. Iterefone yo mu nzu____<br>6. Ifirigo (icuma gikanyisha) ______	HHASSET
1.4.9	Aha mw'uru rugo hari umuntu afise/atunze…?<br>1 – Ego/aragifise/aragitunze<br>0 – Oya ntawuhari/ntaco afise<br>(*andika «**1**» ahari, «**0**» atawuhari*)	1. Ikinga ___<br>2. Umukogote ukwerwa na madudu ___<br>3. Ipikipiki/imoto _____<br>4. Umuduga/ikamyo ____<br>5. Ubwato bw'imoteri ______	HHMBASSET
1.4.10	Murafise ibitungwa?<br>Muroroye ibitungwa ?<br>***Inyishu ari «oya » baza 1.4.12***	|__|1=Ego, |__| 0=Oya	HHLIVESTOCK
1.4.11	Inyishu ari «**Ego**», mufise ibihe bitungwa, muvuge n'igitigiri cavyo	**Ibitungwa** | **Igitigiri**<br>Inka | Intama | Impene | Ingurube | Inkoko (imbata/inkanga) | Ibitwazi | Ibindi: (vuga ivyarivyo) ____ |	NOLIVESTOCK
1.4.12	Murafise isi ndimwa? (Hari uwuba muri runo rugo afise isi ndimwa)	|__|1=Ego, |__| 0=Oya	LANDOWNER
1.4.13	Mufise/afise isi ndimwa ingana gute (ama are-metero kwadrato 100- angahe)	Are/metero kwadrato ……………….	LANDSIZE

Ikigabane ca 2: Amazi n'ibijanye n'isuku

Instruction: *«Munkundire tuyage ibijanye n'ingene muronka amazi yo kunywa, uko muyakoresha hamwe n'ibijanye nuko mutunganya ivy'isuku ngaha mu rugo iwanyu»*

Table 2.1: Amazi hamwe n'itunganwa ry'isuku

	Ibibazo	Inyishu zatanzwe/zishoboka	Inyishu kabanga
2.1	**Amazi mukoresha**		
2.1.1	Amazi munywa/mukoresha aha mu rugo muyakura/muyavoma hehe?	1= irobine/ibomba riri mu rugo<br>2 = amazi y'iriba ritubakiye/ridakingiye<br>3 = amazi y'isoko ryutubakiye/rikingiye<br>4 = amazi yimbwe mu kuzimu hakoreshwa ipompo	WDSOURCE

		5= amazi yo mu mugezi/uruzi/umugende<br>6 = amazi y'imvura ategwa mu gikono	
2.1.2	Amazi mukoresha aha mu rugo (gukinjika, gukaraba ibiganza) muyakura/muyavoma hehe?	1= irobine/ibomba riri mu rugo<br>2 = amazi y'iriba ritubakiye/ridakingiye<br>3 = amazi y'isoko ryutubakiye/rikingiye<br>4 = amazi yimbwe mu kuzimu hakoreshwa ipompo<br>5= amazi yo mu mugezi/uruzi/umugende<br>6 = amazi y'imvura ategwa mu gikono	WSOURCEUZ
2.1.3	Hari ico mukora kugira amazi abe meza imbere yo kuyanywa/kugira ntabatere ingwara?<br>***Inyishu ari «Ego» baza 2.1.4, ari «Oya» baza 2.2.1***	|__|1=Ego, |__| 0=Oya	WTREAT
2.1.4	Mukora iki kugira amazi abe meza imbere yo kuyanywa canke kugira ntabatere ingwara ?	1 = kuyateka/kuyabiza<br>2 = dushiramwo imiti kama/y'ikirundi<br>3 = dushiramwo imiti y'ikizungu<br>4 = turayayungururura<br>5 = turayareka agatonganuka<br>6 = ibindi (vuga ivyarivyo)………………	TREATMTD
**2.2**	**Itunganywa ry'ibijanye n'isuku**		
2.2.1	Murafise akazu ka surwumwe mw'uru rugo ?<br>***Inyishu ari «Ego» bandanya 2.2.2, ari «Oya» simbira kuri 2.2.5***	|__|1=Ego, |__| 0=Oya	HHTOILET
2.2.2	Mukoresha akazu ka surwumwe kameze gate/bwoko ki ?	1 = twalete ikoresha amazi mu gusunika umwanda<br>2 = akazu ka surwumwe gafise idale kandi kubakiye<br>3 = Ikinogo (gifukishije ibiti) kitubakiye<br>4 = Dukoresha indobo	TOILETYP
2.2.3	Hari urundi rugo musangiye ako kazu ka surwumwe canke gakoreshwa n'uru rugo rwonyene?<br>***Inyishu ari «Ego» bandanya, ari «Oya» simbira kuri 2.2.5***	|__|1=Ego, |__| 0=Oya	HSHARTOILT
2.2.4	Musangiye ako kazu ka surwumwe n'ingo zingahe?	1= urugo rumwe 2= ingo zibiri<br>3= ingo zitatu n'izirenga 9 = ntavyo nzi	TOILETSHAR
2.2.5	Ni ryari mukaraba ibiganza? *(Ntusome inyishu zikurikira, nureke uwubungabunga umwana akwishure hanyuma wuzuze* ***1=Ego*** *ku nyishu yanyuma ari nayo ibereye* ***0 = Oya*** *ku zindi nyishu)*		
	Nta na rimwe	1	
	Igihe hariko umwanda/umucafu	2	
	Tuvuye mu kazu ka surwumwe/	3	
	Inyuma yo gusukura umwana	4	
	Imbere yo gutegura imfungurwa	5	
	Imbere yo kugabura imfungurwa	6	
	Imbere yo gufungura	7	
	Imbere yo gufungurira umwana	8	
	Iyo babinyibukije	9	
	Imbere yo kwitaho umurwayi	10	WHNDWASH
	Inyuma yo kwitaho umurwayi	11	

	(1) Imbere n'inyuma yo gutegura imfungurwa (2) Tuvuye mu kazu ka surwumwe/ kwihagarika/inyuma yo gusukura umwana, (3) Imbere yo gufungura canke gufungurira umwana, (4) Imbere n'inyuma yo kwitaho umurwayi	12	
2.2.6	Aha mu rugo iwanyu mukoresha iki mu gukaraba ibiganza ?	0=Amazi meza 1=Amazi meza n'isabuni<br>2= Amazi meza n'umunyota<br>3= Ibindi (vuga ivyarivyo) ……………	WTHANDWASH
2.3	**Guta umwanda mukuru w'umwana atarakwiza imywka ibiri**		
2.3.1	Ubwanyuma uyu mwana (IZINA) yitumye, umwanda wiwe mwawutaye gute ?	01 = umwana yakoresheje akazu ka surwumwe<br>02 = twawutaye mukazu ka surwumwe<br>03 = twawutaye mu kinogo/umufurege<br>04 = Twawutaye muri nyabarega<br>05 = twarawimbiye<br>06 = twawurekeye aho yitumye<br>07 = Ikindi (vuga uko mwawutaye) ……………<br>99 = Ntavyo nzi	STOOLDISPOSAL

GUFUNGURIRA UMWANA, AMAGARA N'IGIKURIRO VYIWE

Instruction*: Nipfuza ko tuganira ibijanye n'ingaburo, amagara y'aumwana n'ingwara akunze kurwara. Ndaza n'ukumupima igikuriro.*

Ikigabane ca 3: Gufungurira umwana *(bibazwa nyina wiwe canke uwusanzwe amubungabunga)*

Table 3.1: Kumwonsa hamwe n'imfungurwa nyunganirabere

	Ibibazo	Inyishu zatanzwe/zishoboka	Inyishu kabanga
3.1.1	Uyu mwana (IZINA) yarigeze yonswa/aronswa?	|__|1=Ego, |__| 0=Oya	CFBREAST
3.1.2	Nimba yaronkejwe, (IZINA) yonkeje amaze umwanya ungana gute avutse ?<br>***Imbere y'uko isaha ihera avutse, andika «00 ». Ari imbere y'amasaha 24, andika amasaha. Ahandi ho andika imisi.***	Akivuka __ ___00<br>Amasaha ___ ___<br>Imisi ____ ___	CFBFEDBIRTH
3.1.3	Mw'iyinga rimwe akivuka, hari ikindi kintu c'ukunywa mwahaye (IZINA) atari amaberebere ?<br>***Inyishu ari «Ego» bandanya, ari «Oya»simbira kuri 3.1.5***	|__|1=Ego, |__| 0=Oya	CFRSEVEN
3.1.4	(IZINA) mwamuhaye iki ?<br>Nta kindi?<br>(***shira akazingi ku numero ijanye n'ikinyobwa bamuhaye***)	1 = ayandi mata (atari amaberebere)<br>2 = amazi<br>3 = amazi y'isukari/iserumu<br>4 = amazi y'ubuzima (SRO)<br>5 = amazi avanze n'ibindi binyobwa<br>6 = amazi y'ivyamwa<br>7 = imfungirwa zikizungu zateguriwe inzoya<br>8 = icayi 9= ikawa<br>10 = ubuki 11 = inzoga kavukire<br>12 = Ibindi (vuga ivyarivyo) ………	CFRTHREDRK

3.1.5	(IZINA) ari kw'ibere/aronswa ubu?<br>***Inyishu ari «Ego» bandanya 3.1.6, ari «Oya», bandaniriza 3.1.6***	|__|1=Ego, |__| 0=Oya	CFBNOW
3.1.6	(IZINA) yarafise amezi angahe igihe yacuka?	Amezi ___	CFAGE
3.1.7	N'iyihe mvo yatumwe acutswa?<br>***(IZINA) yacukijwe kuber'iki ?***	1 = nyina yari yasamye/yibungenze<br>2 = nyina yararwaye<br>3 = nyina yararuhijwe n'ukwonsa<br>4 = umwana yaratanguye gufungura<br>5 = amaberebere yaramurwajije<br>6 = nyina nta maberebere yarafise<br>7 = iyindi (vuga iyariyo) ………	CFBRZNSTOP
3.1.8	Ejo haheze, kuva mugitondo mukivyuka gushika mwijoro muryamye, (IZINA) yonkejwe kangahe ?	Incuro________	CFTIMES
	(Utubazo dukurikira tujanye n'ikintu cambere bahaye umwana kiretse amaberebere : imfungurwa nyunganirabere canke ingaburo yagenewe inzoya/abana bakiri bato).		
3.1.9	Hari iyindi mfungurwa/ngaburo mumaze gutangura guhereza (IZINA) kiretse kumwonsa ?<br>***(IZINA) yaratanguye imfungurwa nyunganirabere ?***	|__|1=Ego, |__| 0=Oya	CFEDOTHER
3.1.10	(IZINA) yari afise imyaka ingahe igihe mwatangure kumuha imfungurwa nyunganirabere ?	Amezi______	CFEDFORMULA

59 **Table: 3.2.Ubwoko/Imirwi y'imfungurwa**

No	Ibibazo	Inyishu zatanzwe/zishoboka	Inyishu kabanga
	N'izihe mfungurwa mwafunguriye/mwagaburiye (IZINA) kuva ejo (haba kumurango canke mwijoro) (***baza ibibazo vyose bikurikira***)	|__|1=Ego |__|0=Oya<br>|__|9=Ntavyo nzi	
3.2.1	Umuceri, umukate, ibitumbura/amandazi, amasaka, uburo canke umusururu (w'umuceri, amasaka, uburo, ibigori)		FCEREAL
3.2.2	Umwungu, ikaroti, ibijumbu bisa n'umuhondo/umutuku imbere)		FVITA
3.2.3	Ibiraya, imyumbati, amateke canke izindi mfungurwa zimeze nk'imizi		FROOTS
3.2.4	Imboga zifise amababi y'urwatsi rutoto rutotahaye		FGREENS
3.2.5	Imyembe, ipapayi, imihwi, amatunda, amabungo, inanasi, imicungwa, ivoca, inkere (bihishiye/bihiye)		FFRUITS
3.2.6	Ikindi camwa icarico cose canke imboga		FVEGS
3.2.7	Inyama y'igitigu, y'ifyigo, y'umutima canke iyindi nyama yo mu nda		FORGANMT
3.2.8	Inyama y'umusoso y'inka, impene, ingurube, intama, inkoko, imbata, n'ibindi		FMEAT
3.2.9	Amagi		FEGG
3.2.10	Ifi, indagara canke ikindi cose kirobwa mu kiyaga/uruzi (uko kiri kwose)		FFISH
3.2.11	Imfungurwa zigizwe n'ibiharage, ubwishaza, inkore canke intete/imbuto		FPULSE
3.2.12	Iforomaji, amata, ikivuguto canke ibindi bikozwe mu mata		FMILK
3.2.13	Amavuta : amamesa,amakoto, amahoke canke ibindi vyose bikomoka mu mavuta		FOIL

3.2.14	Imfungurwa yose irimwo/ikozwe n'isukari :imbombo, ibisuguti, igato, …		FSUGAR
3.2.15	Imivuri/isenene, ibihori, iswa, ibitemvu, ibijogojogo, ibinyabwoya, inkara, ibifyera canke utundi dukoko dutoduto		FINSECTS
3.2.16	Izindi mfungurwa (vuga izarizo)…………………..		FANYOTHER
**3.3. Utundi tubazo**			
3.3.1	Mu ndwi 2 ziheze, haba ku murango canke mwijoro, (IZINA) mwaramugaburiye/yarihereje imfungurwa zongerejwe ingaburo canke zo kumwondagura (umusururu baha abarwaye ingwara yo gufungura nabi-CSB, ifu yongerejwe vitamine/ingaburo, plumpinut) vyatanzwe n'ivuriro, n'amashirahamwe y'abagiraneza	|__|1=Ego, |__| 0=Oya	CSFPFOOD
3.3.2	Ni nde ategurira imfungurwa, agaburira/afungurira (IZINA)?	1 = Uwumubungabunga/nyina<br>2 = Inakuru 3 = Se<br>4 = Mukuru wiwe/mushikiwe<br>5 = Uwundi mwana muto<br>6 = Uwundi (muvuge) ………..	CHDPREFEED
3.3.3	Ugerenanije, uwitaho/uwubungabunga umwana bamarana umwanya ungana gute na (IZINA) ku musi ?	Amasaha_____	CTIMESPENDS

Ikigabane ca 4: Amagara y'umwana n'ingwara akunze kurwara

Baza uwubungabunga umwana kw'afise agakaye kanditswemwo incanco z'umwana. Ibibazo bikurikira biraba ***umwana***.

Table 4.1: Amagara y'umwana

No	Ibibazo	Inyishu zatanzwe/zishoboka	Inyishu kabanga
4.1.1	Murafise agakarata k'incanco ka (IZINA)	|__|1=Ego, |__| 0=Oya	CVACBOOK
4.1.2	(IZINA) yararonse urucanco rwa BCG/IGITUNTU, nukuvuga urushinge bamutera kukuboko canke kurutugu rumukingira ingwara y'igituntu (kandi aho yarutewe hama hasigara inkovu)	1 = Ego, afise n'agakaye<br>2 = Ego, atagira agakaye<br>3 = Oya, afise n'agakaye<br>4 = Oya, atagira agakaye<br>99 = Ntavyo nzi	CBCG
4.1.3	(IZINA) yararonse urucanco rw'amama rwa poliyo, nukuvuga «amama abiri mukanwa» amukingira ingwara y'UBUKANGWE	|__|1=Ego<br>|__|0=Oya<br>|__|99=Ntavyo nzi	CPOLIO
4.1.4	(IZINA) amaze gucandarwa/kuronka urucanco «rw'amama yo mukanwa» rumukingira ingwara y'ubukangwe kangahe	Incuro ……..	CNOPOLIO
4.1.5	(IZINA) yararonse urucanco rwa PENTAVALENT/RURIMWO INCANCO 5, nukuvuga urushinge rwo kwitako rumukingira ingwara zitanu : tetanosi, inkorora y'akanira, ibigoga, mugiga iterwa na *Haemophilus influenzae* y'ubwoko b n'igitigu candukira c'ubwoko bwa B ? Arutererwa rimwe n'amama yo mukanwa amukingira ubukangwe	|__|1=Ego<br>|__|0=Oya<br>|__|99=Ntavyo nzi	CPENTAV
4.1.6	(IZINA) amaze gucandarwa/kuronka urucanco rwa PENTAVALENT/RURIMWO INCANCO 5 rumukingira ingwara 5 kangahe?	Incuro……	CNOPENTAV
4.1.7	(IZINA) yararonse urucanco rw'agasama, nukuvuga urushinge rwo ku kuboko rumukingira ingwara y'AGASAMA na RUBEOLE (afise amezi 9 hamwe na 18)?	|__|1=Ego<br>|__| 0=Oya<br>|__|99=Ntavyo nzi	CMEASLESRU B

4.1.8	(IZINA) amaze gucandarwa/kuronka urucanco rumukingira agasama na RUBEOLE kangahe?	Incuro……	CMEASLESRU BTIME
4.1.9	(IZINA) yararonse urucanco rwa VAROTA (ROTAVIRUS), nukuvuga urucanco rw'amazimazi rumukingira gucibwamo (ROTAVIRUS) ?	|__|1=Ego<br>|__| 0=Oya<br>|__|99=Ntavyo nzi	CROTAVIRUS
4.1.10	(IZINA) amaze gucandarwa/kuronka urucanco rw'amazimazi rumukingira gucibwamo (VAROTA) kangahe?	Incuro……	CROTATIME
4.1.11	(IZINA) yararonse urucanco rwa PNEUMOCOQUE, nukuvuga urushinge rwo kw'itako (ku rindi atari iryo yateweko urwa PENTAVALENT) rumukingira ingwara y'igisonga (pneumonie)	|__|1=Ego<br>|__| 0=Oya<br>|__|99=Ntavyo nzi	CPNEUMO
4.1.12	(IZINA) amaze gucandarwa/kuronka urucanco rumukingira igisonga kangahe?	Nombre de fois……	CPNEUMOTIM E
4.1.13	(IZINA) yararonse vitamini A mu mezi atandatu aheze ?	|__|1=Ego<br>|__| 0=Oya<br>|__|99=Ntavyo nzi	CVITA
4.1.14	(IZINA) yararonse ibinini vy'inzoka mu mezi atandatu aheze ?	|__|1=Ego<br>|__| 0=Oya<br>|__|99=Ntavyo nzi	CDEWORM
4.1.15	(IZINA) n'umwana agira kangahe mu nda ya nyina ?	1 = Imfura, 2 = Kabiri,<br>3 = Gatatu, 4 = Kane,<br>5 = Gatanu, 6 = 6 n'akurenga	CBIRTHORDER
4.1.16	Igihe (IZINA) avuka, yari munini, agereranye, mutomuto canke muto cane?	1 = Munini gose, 2 = Munini, 3 = Agereranye, 4 = mutoyi, 5 = muto cane, 99 = Ntavyo nzi	CBIRTHWGT
4.1.17	(IZINA) yarapimwe ibiro akivuka?<br>***Inyishu ari «Ego», bandanya 4.1.18***	|__|1=Ego<br>|__|0=Oya<br>|__|99=Ntavyo nzi	CWEIGHED
4.1.18	(IZINA) yavukanye ibiro bingahe ?<br><br>***Andika ibiro wisunze agakarata kahari***	1. Kg …… .. vyo kugakarata<br>2. Kg ……. yibutse	CWEIGHT
4.1.19	Twihweje uko incanco zitangwa mu Burundi, (IZINA) yararonse incanco zijanye n'imyaka yiwe ? Ukurikije ivyanditse ku gakarata k'incanco<br>*-Akivuka : BCG-VPO/VPI 0*<br>*- Ukwezi n'igice : VPO/VPI 1- DTC-HépB1+Hib1- VAROTA1 - PCV13 I*<br>*-amezi 2 n'igice : VPO/VPI 2 - DTC-HépB2+Hib2 - VAROTA2- PCV13 II*<br>*-amezi 3 n'igice: VPO/VPI 3 - DTC-HépB3+Hib3 - PCV13 III,*<br>*- amezi 6 : VAROTA 1 -amezi 9 : VAR1*<br>*- amezi 10 : VAROTA 2 -amezi 18 : DTC4 et VAR2*	|__| 1=Ego<br>|__| 2= yaronse incanco zidakwiye<br>|__| 3= Oya nta na rumwe yaronse	CIMMCOMPL

Table 4.2: Ingwara umwana akunze kurwara

Instructions: *«Ubu naho tuganire ibimenyetso canke ingwra uyu mwana wanyu yoba yararwaye mu ndwi zibiri ziheze»*

No	Ibibazo	Inyishu zatanzwe/zishoboka	Inyishu kabanga
4.2.1.	(IZINA) yoba yararwaye mw'izi ndwi zibiri ziheze ?<br>***Inyishu ari «Ego», bandanya ibibazo bikurikira. Ari «Oya» bandaniriza ku gice ca 4.4***	|__|1=Ego, |__| 0=Oya	CILL

4.2.2	N'izihe ngwara /ibihe bimenyetso (IZINA) yoba yararwaye mw'izi ndwi zibiri ziheze? ***Ntusome urwo rutonde rukurikira; reka uwubungabunga umwana adondagure hanyuma wuzuze inyishu aguha***	Mwizo ndwi zibiri ziheze, iyo ngwara wavuze yayirwaye imisi ingahe ?	CILLFRQT
4.2.3	Inkorora	☐☐ imisi	CCOUGH
4.2.4	Guhema nabi/guhema anyarutsa	☐☐ imisi	CFASTBEARTH
4.2.5	Ubushuhe/umucanwa/kururumba MALARIYA	☐☐ imisi	CFEVER
4.2.6	Gucibwamwo (ibisanzwe)	☐☐ imisi	CDIARRH
4.2.7	Gucibwamwo harimwo amaraso	☐☐ imisi	CBLOODIARRH
4.2.8	Izindi ngwra (vuga izarizo) ………………	☐☐ imisi	CILLOTHER

Table 4.3: Ibijanye n'ukwivuza hamwe n'ukuvurwa

No	Ibibazo	Inyishu zatanzwe/zishoboka	Inyishu kabanga
4.3.1.	Igihe (IZINA) yarwara canke akagira ibimenyetso vy'ingwara, mwavyifashemwo mute/mwabigenjeje gute/mwituye nde ? Canke mwamujanye hehe ? *Inyishu ari «**1**» canke «**2**», bandanya akabazo gakurikira, atari izo simbira kuri 4.4.*	1 = Ivuriro/ibitaro (vya leta, abigenga/ abihebeye Imana)<br>2 = Umuremesha kiyago (iCCM)<br>3 = Uwuvuza imiti y'ikirundi<br>4 = Icumba c'amasengesho<br>99 = Ntavyo nzi	TTSEEKING
Ku ngwara/bimenyetso (IZINA) yoba yararwaye mw'izi ndwi zibiri ziheze, yavuwe ate?		Yavuwe uku gukurikira (hisunzwe uko ukuvurwa gutunganijwe n'ubushikiranganji bw'amagara y'abantu):	
4.3.2	Inkorora	Imiti …………………	TTCOUGH
4.3.3	Guhema nabi/guhema anyarutsa	Imiti …………………	TTBREATH
4.3.4	Ubushuhe/umucanwa/kururumba MALARIYA	Imiti …………………	TTFEVER
4.3.5	Gucibwamwo (ibisanzwe)	Imiti …………………	TTDIARRH
4.3.6	Gucibwamwo harimwo amaraso	Imiti …………………	TTBLOODIARRH

4.4. Ibijanye n'ukwivuza hamwe n'ico abaremeshakiyago bunganira

No	Ibibazo	Inyishu zatanzwe/zishoboka	Inyishu kabanga
4.4.1	Iyo mukeneye kwivuza, mukoresha ubuhe buryo bwo kwiyunguruza kugira mushikire ivuriro/ibitaro bibegereye?	1 = Umuduga /ipikipiki (imoto)<br>2 = turipakiza (itagisi, ibisi, imoto)<br>3 = N'amaguru 4 = tugenda n'ikinga<br>5 = Ubundi buryo (vuga ubwaribwo)………	HFACCES
4.4.2	Mukoreshe ubwo buryo bwo kwiyunguruza, bibatwara umwanya ungana gute kugira mushike kw'ivuriro/ibitaro bibegereye ?	1= Iminota 0-30<br>2= Iminota 31-60<br>3= hagati y'isaha 1 n'amasaha 2<br>4= Amasaha abiri n'ayarenga<br>99= Ntavyo nzi	HFTIME
4.4.3	Hari abaremeshakiyago (canke aba Mama muco) basanzwe babaha inyigisho zijanye n'ugufungura neza aha ku mutumba iwanyu?	|__|1=Ego, |__| 0=Oya	CHWNUT

Section 5: Gusuzuma n'ugupima ikama/igabanuka ry'amaraso, malariya hamwe n'inzoka zo munda

Table 5.1: ikama/igabanuka ry'amaraso

Uko bitunganijwe: *«Nipfuza ko tuganira n'ibimenyetso bijanye n'ikama/igabanuka ry'amaraso canke malariya (IZINA) yoba yararwaye mw'iyinga zibizi ziheze. Turamufata amaraso (ku rutoke canke kugitsintsiri kubana bafise hagati y'amezi 6 na 11) kugira dupime kw'ata gabanuka ry'amaraso canke malariya. Ibikoresho bikoreshwa mu gupima amaraso nta ngorane bitera kandi bikoreshwa ku muntu umwe gusa, bihejeje gukoresha duca tubita. Duca tubaha inyishu duheje gupima. Dusanze umwana arwaye malariya canke amaraso yiwe yaragabanutse cane duca tumurungika kw'ivuriro kugira bamufashe uko avurwa. Ibipimo bikorwa mwibanga kandi nta mazina yanyu ashirwa canke azoja ahabona. Vyongeye, ni mwebwe mwenyene muhabwa inyishu z'ibipimo nta wundi azibwirwa.».*

5.1	**Ikama/igabanuka ry'amaraso**	**Inyishu (zishoboka)**	**Inyishu kabanga**
5.1.1	(IZINA) yoba yararwaye ikama/igabanuka ry'amaraso mw'iyinga zibizi ziheze?	|__|1=Ego<br>|__| 0=Oya<br>|__|99=Ntavyo nzi	ANAEMIAHST
5.1.2	Isuzuma ry'ibiganza no mu maso : vyoba vyerekana igabanuka/ikama ry'amaraso (***usuzumye umwana***)	|__|1=Ego, |__| 0=Oya	ANAEMIAPALLOR
5.1.3	Ipimwa ry'urugero rw'amaraso (hemoglobine hakoreshejwe HemoCue)	Amagarama kuri buri litiro**……. (g/l)**	HBSTATUS

Table 5.2 Malariya

5.2.1	(IZINA) yoba yararwaye **malariya mu mezi atandatu aheze** ?<br>*Ntusome ibiranga malariya*, ahubwo baza umuvyeyi ibimenyetso n'uko iyo malariya yapimwe hanyuma uheze wuzuze incuro yayirwaye.*<br>***Inyishu ari «Ego», bandanya akabazo gakurikira. Atar'iyo simbira kuri 5.2.5.***	|__|1=Ego,<br>|__| 0=Oya	MALAREPIZD
5.2.2	Igihe yarwaye malariya, yapimwe hakoreshejwe ubuhe buryo (Bayimupimye gute)?	☐1= basize kukarori/ bapimisha rugagamisha<br>☐2= Igipimo kinyaruka (TDR)<br>☐8= Ntavyo nibuka	MALARSCRN
5.2.3	(IZINA) yoba yararwaye **malariya incuro zingahe** ?	☐1= Rimwe<br>☐2= Incuro 2 - 3<br>☐3= Incuro zirenga 3<br>☐99=Ntavyo nibuka	MALAREPIZNO
5.2.4	(IZINA) yararwaye **malariya ubwanyuma yavuwe n'uwuhe muti**	☐1= Amodiakine na aritezunate (AsAq)/ Aritemeteri na Lumefantrine (AL)<br>☐2= Kinine<br>☐3= Uwundi muti (vuga uwariwo)………..<br>☐99=Ntawo nibuka	MALAREPIZTT
5.2.5	Ipimwa rya malariya (hakoreshejwe TDR)<br>***Fata amaraso (ku rutoke canke kugitsintsiri kumwana bafise hagati y'amezi 6 na 11) ukoreshe amama2 hanyuma nk'uko bitegerezwa***	☐ 1=Arafise malariya<br>☐ 0=Nta malariya afise	MALARSCRN

** **Ibiranga malariya:** kuba yaragize umucanwa/ubushuhe mu masaha 24 aheze, canke ubushuhe $\geq$ 37,5°C hamwe n'igipimo cerekana imigera ya malariya mu maraso.*

5.3	**Gupima umusarani** (Inzoka zo munda)	**Inyishu**	**Inyishu kabanga**
5.3.1	Gupima umusarani : Umwanda mukuru upimwa hakoreshejwe rugagamisha (hamwe n'ubuhinga bwa Kato-Katz)	☐ 1= Ararwaye inzoka<br>☐ 0=Nta nzoka arwaye	HELMTEST
5.3.2	Igipimo cerekana izihe nzoka zo munda	…………………………	PARASITYPE

Ikigabane ca 6. Gupima igikuriro

Uko bigenda*: Umwana apimwe n'abasuzumyi 2 kugira bahinyuze ibipimo (kubipimo batoye) kugitigiri c'amamwe (ubwaguke bw'ukuboko-MUAC, uburebure/igihagararo) canke igitigiri kimwe inyuma y'agakwabu (ibiro).*

No	Isuzuma hamwe n'ibipimo	Inyishu	Inyishu kabanga
6.1.1	Gusuzuma ubuvyimbe bw'ibirenge (ufyonze hagasizagara hicapuye nko kumuhwi uhiye)	☐ 1= Ibirenge biravyimvye<br>☐ 0=Nta buvyimbe bw'ibirenge<br>☐ 8= Ntivyoroshe kwemeza/guhakana	**CBPOSCREEN**
6.1.2	**Ubwaguke bw'ukuboko** (ama cm hakoreshejwe **MUAC**)	Umusuzumyi 1 cm ………. cm ………<br>Umusuzumyi 2 cm ………. cm ………	MUAC
6.1.3	**Uburemere/ibiro** (Kg)	Umusuzumyi 1 Kg ………. Kg ………<br>Umusuzumyi 2 Kg ………. Kg ………	WEIGHT
6.1.4	**Uburebure/Igihagararo (cm)**<br>Uko bigenda: *abana batarakwiza imyaka 2 bapimwa baryamitswe kugipimisho (uburebure) abamaze gukwiza imyaka ibiri bapimwa bahagaze*	Umusuzumyi 1 ……….cm ………cm<br>Umusuzumyi 2 ……….cm ………cm	LENGTH
		Umusuzumyi 1 ……….cm ………cm<br>Umusuzumyi 2 ……….cm ………cm	HEIGHT

	Uwakoresheje ikiganiro/Umusuzumyi 1	Uwakoresheje ikiganiro/Umusuzumyi 2	Umugenduzi	Uwushira inyishu kabanga mu nyabwonko #1	Uwushira inyishu kabanga mu nyabwonko #2
Code/ Initiales	|__|__|/______	|__|__|/______	|__|__|/_______	|__|__|/______	|__|__|/_______
Itariki	|__|__| / |__|__| / 20|__|__|	|__|__| / |__|__| / 20|__|__|	|__|__| / |__|__| / 20|__|__|	|__|__| / |__|__| / 20|__|__|	|__|__| / |__|__| / 20|__|__|
