# Supplementary material for: Prevalence and factors associated with anaemia in children aged 6–24 months living a high malaria transmission setting in Burundi
Source: PLoS One. 2022 Sep 2;17(9):e0273651. doi: 10.1371/journal.pone.0273651 (PMC9439186; doi:10.1371/journal.pone.0273651)
Supplement: Attachment — Submitted filename: Response to Reviewers.docx [file pone.0273651.s004.docx]

February 10, 2022

To: Academic Editor

PLOS ONE

Reference number: PONE-D-21-22573

Thank you very much for the careful consideration of our manuscript and for inviting us to re-submit our revised manuscript (based on the Reviewers' comments) entitled: “Prevalence and factors associated with anaemia in children aged 6-24 months living a high malaria transmission setting in Burundi ” Below are point by point responses to the reviewers’ comments. We are re-submitting a revised version of the manuscript that addresses the points raised during the review process. We look forward to your favourable consideration of this re-submission.

POINTS BY POINTS RESPONSES TO THE REVIEWERS’ COMMENTS

1. Please ensure that your manuscript meets PLOS ONE's style requirements, including those for file naming. The PLOS ONE style templates can be found at 
<https://journals.plos.org/plosone/s/file?id=wjVg/PLOSOne_formatting_sample_main_body.pdf> and 
<https://journals.plos.org/plosone/s/file?id=ba62/PLOSOne_formatting_sample_title_authors_affiliations.pdf>

The revised manuscript was adapted to PLOS ONE’s style requirements

2. Please include additional information regarding the survey or questionnaire used in the study and ensure that you have provided sufficient details that others could replicate the analyses. For instance, if you developed a questionnaire as part of this study and it is not under a copyright more restrictive than CC-BY, please include a copy, in both the original language and English, as Supporting Information.

Study questionnaire in English is available on protocols.io: dx.doi.org/10.17504/protocols.io.b4ryqv7w

Study questionnaire in local language (Kirundi) is available as attached file under materials section on protocols.io: dx.doi.org/10.17504/protocols.io.b4vxqw7n

3. We note that you have stated that you will provide repository information for your data at acceptance. Should your manuscript be accepted for publication, we will hold it until you provide the relevant accession numbers or DOIs necessary to access your data. If you wish to make changes to your Data Availability statement, please describe these changes in your cover letter and we will update your Data Availability statement to reflect the information you provide.

Data of the study involve indirect identifiers (names initials, sex, age, location) and may risk the identification of study participants. Ethical restrictions may be applied on the data supporting this study in the interest of protecting confidential participant information. To access data, interested and qualified researchers may submit requests to the School of Medicine Ethics Committee at the College of Health Sciences, Makerere University ([rresearch9@gmail.com](mailto:rresearch9@gmail.com)), can also submit demands to the current chairperson of the committee, Prof. Ponsianoo Ocama ([ponsiano.ocama@gmail.com](mailto:ponsiano.ocama@gmail.com)) or to the Institutional Ethical Committee on Health Research of the Faculty of Medicine, University of Burundi ([faculte_medecine_ub@yahoo.fr](mailto:faculte_medecine_ub@yahoo.fr)).

4. Your abstract cannot contain citations. Please only include citations in the body text of the manuscript, and ensure that they remain in ascending numerical order on first mention.

Ok, no citations in abstract.

5. Please include a copy of Tables 1 and 2 which you refer to in your text on pages 11 and 12.

Table 1 on pages 12-13 and Table 2 on pages 13

6. Please review your reference list to ensure that it is complete and correct. If you have cited papers that have been retracted, please include the rationale for doing so in the manuscript text, or remove these references and replace them with relevant current references. Any changes to the reference list should be mentioned in the rebuttal letter that accompanies your revised manuscript. If you need to cite a retracted article, indicate the article’s retracted status in the References list and also include a citation and full reference for the retraction notice.

Reference list corrected and completed.

Major comments:

1. Provide more details of systematic sampling procedure referred to in Study Population section (Line 86)

Response:

Using the computed sample size of 492 participants, and the registered total number of children aged 6-24 months in Mukenke Health center catchment area (n=1116), we calculated the sampling interval by dividing accessible population by the sample size (1116/492=2,27 rounded at 2). Using the community health workers (CHW) registers in each village (the register lists the children and their households), we selected households to participate in the study using an interval of 2 as listed in the register. We selected only one participant (child aged 6-24 months) per household using inclusion and exclusion criteria (page 6 lines 89-99).

2. Line 168 Data Management and analysis. A little more detail about the cluster adjustment for administrative hills level.

Response:

We anticipate that clustering exists at the administrative hills level and we adjusted for this by including administrative hills as a covariate to the final model. The final adjusted model of significant covariates was

Logit (anaemia) = 1.89 + 1.08 Budahunga +1.01 Mukenke+ 1.40 No deworming	+0.81 Tertiary Ed -0.81 poor SES -0.96 Middle SES- 0.86 Rich SES

[Stata command: logit anaemia i.chsex ib2.ChageGpe ib2.rezhill ib1.BreastFdSt ib2.MothAge ib1.Deworming i.CgChrelat i.Under5sibl i.CgEducGp i.SES3quintile3 ib1.csfpfood Stunting Wasting2 Underweight i.malarscrn, vce(cluster rezhill) ]

3. Line 177. Sentence appears to be incomplete

All variables showing an association at 20% significance level at bivariate analysis and potential confounders were considered for inclusion into the multivariable logistic regression model (page 10 lines 183-184).

4. Line 183 Describe what criterion was used for eliminating models. For instance, AIC, BIC etc,

Response:

For eliminating models, we used Akaike’s Information Criteria (AIC) and Bayesian Information Criteria (BIC). Smaller values of AIC and BIC indicating better model fitting (page 10 lines 192-194).

Minor comments:

5. Abstract/Conclusion. Line 25 and 26. Please revise sentence ‘

Sentence revised (page 3 line 27)

6. Introduction: Line 48. Change county to country

county changed to country (page 4 line 49)

7. Line 123 Study Procedures. Change plasmodium to Plasmodium

plasmodium changed to Plasmodium (page 8 line 129)

8. Line 158. Change underweighted to underweight

underweighted changed to underweight (page 9 line 164)

9. Results Line 221 Change table 2 to Table 2

table 2 changed to Table 2 (page 13 line 230)

10. Line 228- 229 MUKENKE, BUDAHUNGA and BUTEGANA should all be written in Sentence case

MUKENKE, BUDAHUNGA and BUTEGANA written in Sentence case: Mukenke, Budahunga and Butegana (page 15 lines 237- 238)

11. Line 304, 306 and 341. Same comments as above

Sentence case of Mukenke and Budahunga (page 18 lines 312), Butegana (page 18 line 314), Mukenke (page 20 line 349)

12. Conclusions Line 348. Delete in

in deleted (page 20 line 357)
