# Supplementary material for: Prevalence and factors associated with anaemia in children aged 6–24 months living a high malaria transmission setting in Burundi
Source: PLoS One. 2022 Sep 2;17(9):e0273651. doi: 10.1371/journal.pone.0273651 (PMC9439186; doi:10.1371/journal.pone.0273651)
Supplement: Attachment — Submitted filename: Plos_anaimia_manuscript.docx [file pone.0273651.s005.docx]

**Dear Editor,**

I would like to say thank you very much for inviting me to review this manuscript. The authors investigating prevalence of anaemia and associated risk factors among young children in malaria high endemic setting in Burundi. The findings will be used to initiate anaemia reducing measures to avert the long-term effect of growing children. However, I have the following points to be cleared and addressed. The authors mentioned that the hemoglobin level was adjusted for altitude but how the altitude data from the households is collected (data is collected in paper). The other main concern of this manuscript is the deworming and its association with anaemia. It is doubtful whether children less than 2 years were targeted in deworming program or not. Since all children were not infected by any helminthic infections, the contribution of deworming to the anaemia seen as chance effect. The other specific concerns are mentioned below per sections of the article

**Abstract:**

1. Why high malaria transmission setting is selected as long as the objective of the study was not to estimate the effect of malaria on anaemia of the young children?
2. How stool specimen collected from the children was diagnosed should be mentioned here?
3. In the result part, is the moderate anaemia (59.2%) from the total studied children or out of those anaemic? It is not clear. Either rephrase or put the number
4. Are those less than 2 years are targeted for the deworming in your national program? If so, what are the deworming drugs used for and what diseases are targeted?
5. In line 21 through 23, the authors indicated that SES is inversely related to anaemia (poor, middle and reach). What is the reference category where the OR should be 1?
6. The conclusion and the result are not in alignment, mainly nothing is said in the result about malaria but recommended to improve malaria prevention measures.

**Introduction:**

1. Definition of anaemia (line 33-35), it is not only the number of red blood cells but also the hemoglobin concentration also maters in adequate number of red blood cells in the body. It needs redefining anaemia again.
2. The introduction is with minimum literature on the burden of anaemia and factors affecting the its prevalence. Please include more literature.

**Methods:**

1. As to me, the malaria, anaemia and nutritional status of children related information in the study area should be taken back to the background section. It is enough to mention the demographic variables and the environmental factors.
2. in line 84-87, the authors indicated that community health workers registry (frame) were used to select the participating child by systematic sampling technique. This implies, data collectors go to the households with eligible child in the household. In contrast to this, the authors mentioned that in case of absence of young child in the household, the nearest household is approached. This has to be corrected based on what is practically done in the survey
3. line 95-96 indicates that sickle cell anaemia was not concern of the study. Why that is so and how did you checked whether the child is with sickle cell anaemia or not? This has to be mentioned clearly
4. what are the specific variables included to generate the wealth index (Line 161-62)? Are the variable all numeric (i.e., quantitative)? If all variable are quantitative, go with the PCA, qualitative only use the MCA and mixed type, it is advisable to use multiple factor analysis.
5. In line 165 through 167, and also in the abstract section, hemoglobin level is adjusted for altitude but the data is collected by questionnaire and double entered into a software. How you get the altitude of the household for the children involved in the study.
6. The sample size estimation part should be separated from the analysis (line 171-175). The hypothesis or the research question should be placed at the end of the introduction section of the manuscript.
7. The statement on line 177 is incomplete. Make it complete
8. Line 177-187 is part of the result section. Why multivariable analysis was made is to take care of the confounding effect.
9. one of the factors identified was the location of the study, implying that the anaemia was clustered and thus, analysis shall consider this into account by using the location/village as random variable than taking it as fixed variable in the analysis

**Results:**

1. in line 219-221, malaria association with anaemia was slightly higher in spite of statistically significant association? Why slightly…since the p-vale indicates significant
2. it is better is the OR is presented with 95%CI than only the p-value in the text of the result section
3. socioeconomic status or social economic status is correct?
4. absence of any parasitic infections from the kato-katz technique indicates that the young children were not at risk for such infections. The association between deworming and anaemia seems chance effect than true effect due to not dewormed. It may be solved if the clustered nature of the date is taken into account during the analysis. Furthermore, I do not think that there are drug formulations for mass campaign for children age less than 2 years. Check this with your national deworming program.
5. In table 2, malaria test result is significant if we look the p- value but 95% CI indicates insignificant. Then, how this is calculated? Check your analysis.
6. Unadjusted versus Crude? Interchangeably used. It is better if you consistently use the crude OR (tables)

**Discussion**

1. I think the discussion should not be crowded by numbers for comparison. Such numbers on the prevalence of different study findings shall be presented in the introduction section. And make the comparisons based on the level of significance and looking your confidence intervals than putting the numbers. The same thing is true for effect size of the factors.
2. As to me parts from line 289-299 are out of the scope of this manuscript and shall be removed.
3. Your discussion from line 311 to 321 is about heterogeneity of anaemia with geographic location suggesting analysis to account clustered nature of the data
4. For the general conclusion, look the comment on the conclusion section of the abstract.
