# Supplementary material for: Prevalence and factors associated with anaemia in children aged 6–24 months living a high malaria transmission setting in Burundi
Source: PLoS One. 2022 Sep 2;17(9):e0273651. doi: 10.1371/journal.pone.0273651 (PMC9439186; doi:10.1371/journal.pone.0273651)
Supplement: Attachment — Submitted filename: Response to Reviewers.docx [file pone.0273651.s006.docx]

May 4, 2022

To: The Editor

PLOS ONE

Reference number: PONE-D-21-22573R1

Response to review comments for the manuscript entitled: “Prevalence and factors associated with anaemia in children aged 6-24 months living a high malaria transmission setting in Burundi” Ref: PONE-D-21-22573R1

Thank you very much for the careful consideration of our manuscript and for inviting us to re-submit our revised manuscript (based on the Reviewers' comments) entitled: “Prevalence and factors associated with anaemia in children aged 6-24 months living a high malaria transmission setting in Burundi ” Below are point by point responses to the reviewers’ comments. We are re-submitting a revised version of the manuscript that addresses the points raised during the second review. We look forward to your favourable consideration of this re-submission.

POINTS BY POINTS RESPONSES TO THE REVIEWERS’ COMMENTS

**Abstract:**

**Comment 1**: Why high malaria transmission setting is selected as long as the objective of the study was not to estimate the effect of malaria on anaemia of the young children?

**Response:** This manuscript presents the baseline data of an on-going stud evaluating the effect of Micronutrient supplementation on anaemia and cognition among children in high malaria transmission setting in Burundi. Given the source of data, the setting is dictated by the primary study. This clarification has been included both in the abstract and main text of the manuscript (Lines 6-9 and lines 87 -89).

**Comment 2**: How stool specimen collected from the children was diagnosed should be mentioned here?

**Response:** A universal laboratory container, a plastic bag, a clean non-disinfectant impregnated disposable towel and tissue paper were provided to the primary caregivers of the children enrolled and the care givers were asked to collect morning stool sample which was used to examine for presence of helminth infections. This detail is not provided in the abstract given the word limit, but is provided in the methodology sections (Lines 161 - 163). No change has been made to the manuscript.

**Comment 3**: In the result part, is the moderate anaemia (59.2%) from the total studied children or out of those anaemic? It is not clear. Either rephrase or put the number

Response: The 59.2% is the proportion of the 370 participants with aneamia that were defined as having moderate anaemia. This has been made clear in the abstract (line 20).

**Comment 4**: Are those less than 2 years are targeted for the deworming in your national program? If so, what are the deworming drugs used for and what diseases are targeted?

**Response**: Deworming programs in Burundi target children 1 year – 14 years and are conducted as part of the nationwide health campaign. Albendazole is the drug used for deworming the campaigns. The study population included children 6 months to 24 months and data on routine deworming was captured in this study as detailed in the manuscript (line 128-129).

**Comment 5**: In line 21 through 23, the authors indicated that SES is inversely related to anaemia (poor, middle and rich). What is the reference category where the OR should be 1?

**Response:** The reference category for social economic status (SES) is ‘Very poor SES’. The reference category has been included in the abstract (line 28), and details are presented in Table 2.

**Comment 6:** The conclusion and the result are not in alignment, mainly nothing is said in the result about malaria but recommended to improve malaria prevention measures.

**Response:** The prevalence of malaria was estimated by rapid diagnosis tests 12.5% (now included in the abstract, line 18-19). Although the association between malaria and anaemia was not statistically significant, children with malaria had 2.5 times higher odds of having anaemia than those without malaria and since previous literature has shown this to be an important predictor for anaemia, we recommend its prevention in this high transmission setting.

**Introduction:**

**Comment 1**: Definition of anaemia (line 33-35), it is not only the number of red blood cells but also the hemoglobin concentration also maters in adequate number of red blood cells in the body. It needs redefining anaemia again.

**Response:** Anaemia has been redefined as a condition in which the number of red blood cells is reduced or the haemoglobin concentration within them is lower than normal, consequently affecting their capacity to carry sufficient oxygen is to meet the body’s physiologic needs (line 37 - 39).

**Comment 2:** The introduction is with minimum literature on the burden of anaemia and factors affecting the its prevalence. Please include more literature.

**Response:** The introduction has been expanded to include some more literature on anaemia and factors that have been associated with it (line 39-45).

**Methods:**

**Comment 1**: As to me, the malaria, anaemia and nutritional status of children related information in the study area should be taken back to the background section. It is enough to mention the demographic variables and the environmental factors.

**Response:** Except when describing the study setting in the methods section, all other information on malaria, anaemia and nutritional status has been included in the introduction.

**Comment 2**: In line 84-87, the authors indicated that community health workers registry (frame) were used to select the participating child by systematic sampling technique. This implies, data collectors go to the households with eligible child in the household. In contrast to this, the authors mentioned that in case of absence of young child in the household, the nearest household is approached. This has to be corrected based on what is practically done in the survey

**Response:** Although the community health workers registry was used to identify households with children aged 6 -24 months, these registers are not routinely updated and, in some cases, when households were approached, respondents in the age-group of interest were missing requiring their replacement.

**Comment** 3: line 95-96 indicates that sickle cell anaemia was not concern of the study. Why that is so and how did you checked whether the child is with sickle cell anaemia or not? This has to be mentioned clearly.

**Response:** The exclusion of sickle cell anaemia was dictated by the primary source of the data which aimed at evaluating the effect of micronutrient supplementation and some of the micro-supplements were contra-indicated in children with sickle cell anaemia.

**Comment 4:** what are the specific variables included to generate the wealth index (Line 161-62)? Are the variable all numeric (i.e., quantitative)? If all variable are quantitative, go with the PCA, qualitative only use the MCA and mixed type, it is advisable to use multiple factor analysis.

**Response:** Variables used in the SES included: household head earning income, house ownership, house wall, main source of energy for cooking, main type of floor and main source of drinking water. This detail is included in the manuscript (line 186-189).

**Comment 5:** In line 165 through 167, and also in the abstract section, hemoglobin level is adjusted for altitude but the data is collected by questionnaire and double entered into a software. How you get the altitude of the household for the children involved in the study.

**Response:** We applied altitude adjustment on haemoglobin measured values for Mukenke altitude based on the midpoint of altitude range (1403.5 m for the 1331−1476 range), we used 1989 MMWR equation for Hb adjustment (g/L) = [(−0.032 × (altitude × 0.0032808) + 0.022 × (altitude × 0.0032808)2) × 10] = -3.20 g/L (line 147-150).

**Comment 6:** The sample size estimation part should be separated from the analysis (line 171-175). The hypothesis or the research question should be placed at the end of the introduction section of the manuscript.

**Response:** This has been rectified (line 71-72)

**Comment 7**: The statement on line 177 is incomplete. Make it complete

**Response:** The statement has been rectified and currently reads as follows: **‘**Although child sex, breastfeeding status, relationship to primary care giver, number of under five siblings <5 years of age, stunting status were not showing an association at 20% significance level at bivariate analysis, we also included them into the multivariable logistic regression final model on basis of apriori knowledge’. (line 206-208)

**Comment 8**: Line 177-187 is part of the result section. Why multivariable analysis was made is to take care of the confounding effect.

**Response:** The analysis is considered as part of the methods sections and details of what was included in the model at multivariate analysis including variables with p-value <0.2, factors found as confounders, and factors that are known to confound the association are presented in the sections as part of what constituted the final model. The estimates from the multivariate analysis/ final model are then presented in the results section for each factor.

**Results:**

**Comment 1:** in line 219-221, malaria association with anaemia was slightly higher in spite of statistically significant association? Why slightly…since the p-vale indicates significant.

**Response:** The prevalence of anaemia was also higher in children who had a positive mRDT test than those that were negative (87.1% versus 72.5%, p-value= 0.017) for bivariate analysis (line 244-246).

**Comment 2**: it is better is the OR is presented with 95% CI than only the p-value in the text of the result section

**Response:** We agree with the comment and all odds ratios have been presented with 95% CI:

Our study showed that children from Mukenke and Budahunga had almost 3 times higher odds of having anaemia than children from Butegana (aOR=2.76; 95% CI: 1.86-4. 08 and aOR=2.95; 95% CI: 2.70-3.21 respectively). Increasing social economic status was associated with reduced odds of having anaemia (aOR=0.44; 95% CI: 0.23-0.83 for children from poor households, aOR=0.27; 95% CI: 0.16-0.90 for children from middle socioeconomic status households and aOR=0.42; 95% CI: 0.25-0.72 for children from rich households) (line 255-259).

Children who didn’t take deworming medication in the last 6 months had higher odds of having anaemia compared to those who took deworming medication (aOR=4.06; 95% CI: 2.44-6.76; p-value <0.001). Children whose primary care giver have a tertiary education level had less odds of having anaemia (aOR=0.45; 95% CI: 0.25-0.72; p-value <0.001) than others (line 260-262).

**Comment 3**: Socioeconomic status or social economic status is correct?

**Response:** We used socioeconomic status: any measure which attempts to classify individuals, families, or households in terms of indicators such as occupation, income, and education. ... (John Scott and Gordon Marshall. Oxford Dictionary of Sociology 3 ed, Oxford University Press, ISBN-13: 9780199533008).

**Comment 4**: Absence of any parasitic infections from the kato-katz technique indicates that the young children were not at risk for such infections. The association between deworming and anaemia seems chance effect than true effect due to not dewormed. It may be solved if the clustered nature of the date is taken into account during the analysis. Furthermore, I do not think that there are drug formulations for mass campaign for children age less than 2 years. Check this with your national deworming program.

**Response:** We already mentioned that children between 1 and 14 years got albendazole during nationwide health campaign for child survival (line 128-129).

Our results revealed that not receiving deworming medication (albendazole for children between 1 to 14) during countrywide health campaign for child survival was statistically significant associated with anaemia (adjusted Odd ratio = 4.06, 95% CI 2.01-8.21, p<0.001), it maybe explained by the fact that children aged 14 to 23 months received albendazole during December 2019 deworming campaign (they were part of the targeted population of 1 to 14 years) , absence of any parasitic infections from the kato-katz technique could be also a result of the deworming campaign.

The first round of health campaign for child survival took place from 25^th^ to 28^th^ June 2019 (OMS. Lancement à Karuzi de la première édition de la Semaine Santé Mère-Enfant 2019 available on <https://www.afro.who.int/fr/news/lancement-karuzi-de-la-premiere-edition-de-la-semaine-sante-mere-enfant-2019> ) and the second round in December 2019 while the first round of 2020 health campaign for child survival took place in June 2020 (UNICEF. For every child, vitamin A and deworming tablets. Health campaign in Burundi targets over 5 million children available on <https://www.unicef.org/burundi/stories/every-child-vitamin-and-deworming-tablets> ).

**Comment 5**: In table 2, malaria test result is significant if we look the p- value but 95% CI indicates insignificant. Then, how this is calculated? Check your analysis.

**Response:** We checked our analysis and got same results, that’s why we mentioned that **‘**Even if the p-value was not significant, our bivariate analysis showed that children with a positive malaria test have more than two times the odds of a having anaemia than children with a negative test (OR=2.56; 95% CI:0.18-5.55; p-value 0.141)’ (line 286-288).

**Comment 6**: Unadjusted versus Crude? Interchangeably used. It is better if you consistently use the crude OR (tables)

**Response:** We changed in Table 2 and used crude odds ratio.

**Discussion**

**Comment 1**: I think the discussion should not be crowded by numbers for comparison. Such numbers on the prevalence of different study findings shall be presented in the introduction section. And make the comparisons based on the level of significance and looking your confidence intervals than putting the numbers. The same thing is true for effect size of the factors.

**Response:** We made comparisons as suggested.

Tesema et al in a study on prevalence and determinants of severity levels of anaemia among children aged 6–59 months in sub-Saharan Africa found that Children from poorest, poorer, middle and richer household wealth were 1.39 times [AOR = 1.39, 95% CI: 1.33, 1.45], 1.32 times [AOR = 1.32, 95% CI: 1.26, 1.37], 1.20 times [AOR = 1.20, 95% CI: 1.15, 1.25], and 1.15 times [AOR = 1.15, 95% CI: 1.11, 1.20] higher odds of higher level of anemia compared to children from the richest household wealth, respectively (line 327-333).

Children whose mothers had a tertiary education level were less likely to be anaemic (aOR: 045, 95% CI 0.25-0.72). For Tesema et al children whose mother education level had 1.73 times (AOR = 1.73, 95% CI: 1.60, 1.86) at no formal education, 1.39 times (AOR = 1.39, 95% CI: 1.29, 1.50) at primary education, and 1.27 times (AOR = 1.27, 95% CI: 1.18, 1.36) at secondary education level than children whose mother had a higher level of education (line 351-355).

Watthanakulpanich et al, in their study in Thailand reported that anaemic status of the hookworm positive group significantly improved by 2 months after deworming of the hookworm-infected children (Hb= 12.1, 95% CI 5.1-19.2) and to became comparable with the helminth-free control group(Hb= 12.0, 95% CI 9.2-15.7) (line 361-364).

On the other hand, a study on effect of deworming on school-aged children’s physical fitness, cognition and clinical parameters in a malaria-helminth co-endemic area of Ivory Cost revealed that deworming showed no effect on haemoglobin levels and anaemia. The mean Hb levels did not differ significantly between surveys (baseline, 120.4 g/l; follow-up, 119.9 g/l; p = 0.631) and the overall prevalence of anaemia remained unchanged (baseline, 34.3%; follow-up, 34.3%) (line 366-369).

**Comment 2**: As to me parts from line 289-299 are out of the scope of this manuscript and shall be removed.

**Response:** Parts from line 289-299 (line 320-334 in revised manuscript) are explanations of High prevalence of anaemia in the very young children.

**Comment 3**: Your discussion from line 311 to 321 is about heterogeneity of anaemia with geographic location suggesting analysis to account clustered nature of the data.

**Response:** we adjusted for administrative hills and included this cluster adjustment in the final model.

**Comment 4**: For the general conclusion, look the comment on the conclusion section of the abstract.

**Response:** As previously mentioned in response to comment 6 of the abstract, ‘The prevalence of malaria was estimated by rapid diagnosis tests 12.5%’ (included in the abstract, line 18-19). Although the association between malaria and anaemia was not statistically significant, children with malaria had 2.5 times higher odds of having anaemia than those without malaria (line 286-288) and since previous literature has shown this to be an important predictor for anaemia, we recommend its prevention in this high transmission setting.
