# Supplementary material for: Prevalence and factors associated with anaemia in children aged 6–24 months living a high malaria transmission setting in Burundi
Source: PLoS One. 2022 Sep 2;17(9):e0273651. doi: 10.1371/journal.pone.0273651 (PMC9439186; doi:10.1371/journal.pone.0273651)
Supplement: Attachment — Submitted filename: review round 2.docx [file pone.0273651.s007.docx]

**Dear Prof. Mary Hamer Hodges,**

Thank you for inviting me to review the manuscript entitled “**Prevalence and factors associated with anaemia in children aged 6-24 months living a high malaria transmission setting in Burundi**”. The authors have addressed most of the comments given in the 1^st^ round of my review except very few. If the authors address the following comments, the manuscript can be accepted. The comment numbers are the comments indicated in my previous review comment.

**Methods**

**Comment 4:** what are the specific variables included to generate the wealth index (Line 161-

62)? Are the variable all numeric (i.e., quantitative)? If all variable are quantitative, go with

the PCA, qualitative only use the MCA and mixed type, it is advisable to use multiple factor

analysis.

This part of my comment is not well addressed. PCA is used for dimension reduction of continuous variables, multiple correspondence analysis (MCA) for categorical data and if the variables used to reduce dimension of the data is mixed (i.e both categorical and continuous) like the case in the current manuscript, multiple factor analysis is used. Thus, I recommend to use MFA instead of PCA in the current manuscript.

**Comment 5:** In line 165 through 167, and also in the abstract section, hemoglobin level is

adjusted for altitude but the data is collected by questionnaire and double entered into a

software. How you get the altitude of the household for the children involved in the study.

The response provided by the authors for this comment is not satisfactory. I recommend adjusting hemoglobin for altitude based on reference Haemoglobin concentrations for the diagnosis of anaemia and assessment of severity published by WHO available at the following URL.

https://apps.who.int/iris/bitstream/handle/10665/85839/WHO_NMH_NHD_MNM_11.1_eng.pdf

**Results**

**Comment 4**: Absence of any parasitic infections from the kato-katz technique indicates that

the young children were not at risk for such infections. The association between deworming

and anaemia seems chance effect than true effect due to not dewormed.

Is there any causal pathway that deworming can increase the hemoglobin concentration in the absence of any parasitic infections? I think it is not the deworming drug that influenced the hemoglobin concertation, rather it may be the supplementations given to the children during the deworming campaign. If this was not the case, I think it is better to remove deworming from the analysis.

**Discussion**

**Comment 1**: I think the discussion should not be crowded by numbers for comparison. Such

numbers on the prevalence of different study findings shall be presented in the introduction

section. And make the comparisons based on the level of significance and looking your

confidence intervals than putting the numbers. The same thing is true for effect size of the

factors. This comment is misunderstood by the authors. I recommend not to use the numbers which are indicated in the introduction and result section of the manuscript. Remove the odds ration and its 95% CI in the discussion section.
