# Supplementary material for: Prevalence and factors associated with anaemia in children aged 6–24 months living a high malaria transmission setting in Burundi
Source: PLoS One. 2022 Sep 2;17(9):e0273651. doi: 10.1371/journal.pone.0273651 (PMC9439186; doi:10.1371/journal.pone.0273651)
Supplement: Attachment — Submitted filename: Response to reviewer2.2nd round_PlosOne_anaemia manuscript.docx [file pone.0273651.s008.docx]

July 28, 2022

To: The Editor

PLOS ONE

Reference number: PONE-D-21-22573R2

**Response to review comments for the manuscript entitled: “Prevalence and factors associated with anaemia in children aged 6-24 months living a high malaria transmission setting in Burundi” Ref: PONE-D-21-22573R2.**

Thank you very much for the careful consideration of our manuscript and for inviting us to re-submit our revised manuscript (based on the second round of Reviewers' comments) entitled: “Prevalence and factors associated with anaemia in children aged 6-24 months living a high malaria transmission setting in Burundi” Below are point by point responses to the reviewers’ comments. We are re-submitting a revised version of the manuscript that addresses the points raised during the second review. We look forward to your favourable consideration of this re-submission.

POINTS BY POINTS RESPONSES TO THE REVIEWERS’ COMMENTS

**Methods**

**Comment 1:** what are the specific variables included to generate the wealth index (Line 161-

62)? Are the variable all numeric (i.e., quantitative)? If all variable are quantitative, go with

the PCA, qualitative only use the MCA and mixed type, it is advisable to use multiple factor

analysis. **Our response**: “Variables used in the SES included: household head earning income, house ownership, house wall, main source of energy for cooking, main type of floor and main source of drinking water. This detail is included in the manuscript (line 186-189). ”: **Comment to response**: This part of my comment is not well addressed. PCA is used for dimension reduction of continuous variables, multiple correspondence analysis (MCA) for categorical data and if the variables used to reduce dimension of the data is mixed (i.e both categorical and continuous) like the case in the current manuscript, multiple factor analysis is used. Thus, I recommend to use MFA instead of PCA in the current manuscript.

**Response:** *Thank you for this in-put, we have now used the MFA instead of PCA to generate the wealth index. From the new analysis, socioeconomic status was no longer significant in the final model and has been dropped. The updated results are presented in Table 1 (Page 13-14) and Table 2 (Page 15) as well as the text (line 200-1203).*

**Comment 2:** In line 165 through 167, and also in the abstract section, hemoglobin level is adjusted for altitude but the data is collected by questionnaire and double entered into a

software. How you get the altitude of the household for the children involved in the study. **Our response**: We applied altitude adjustment on haemoglobin measured values for Mukenke altitude based on the midpoint of altitude range (1403.5 m for the 1331−1476 range), we used 1989 MMWR equation for Hb adjustment (g/L) = [(−0.032 × (altitude × 0.0032808) + 0.022 × (altitude × 0.0032808)2) × 10] = -3.20 g/L (line 147-150). **Comment to response**: The response provided by the authors for this comment is not satisfactory. I recommend adjusting hemoglobin for altitude based on reference. Haemoglobin concentrations for the diagnosis of anaemia and assessment of severity published by WHO available at the following URL.

<https://apps.who.int/iris/bitstream/handle/10665/85839/WHO_NMH_NHD_MNM_11.1_eng.pdf?sequence=22&isAllowed=y>

**Response:** *WHO-based cutoffs for Children 6 - 59 months of age are: Non- anaemia: 110 g/l or higher, 100-109 g/l for mild, 70-99 g/l for moderate and lower than 70 g/l for severe anaemia. While altitude adjustments to measured haemoglobin concentrations is -2g/l for 1000m metres above sea level and -5g/l for 1500 metres above sea level. In this study we didn’t not collect the altitude estimates for the homes of each child, however, we used the midpoint altitude estimates of the study area (Mukenke altitude ranges between 1331−1476 and we used 1403.5 m for the average estimate)as reported in elevation map^[1]^. We used the CDC formula published in 1989 MMWR “equation for Hb adjustment (g/L) = [(−0.032 × (altitude × 0.0032808) + 0.022 × (altitude × 0.0032808)2) × 10]” = -3.20 g/L ^[2]^,^[3]^. This detail is included in the manuscript, line 160-164****.***

**Comment 3**: Absence of any parasitic infections from the kato-katz technique indicates that

the young children were not at risk for such infections. The association between deworming

and anaemia seems chance effect than true effect due to not dewormed. Is there any causal pathway that deworming can increase the hemoglobin concentration in the absence of any parasitic infections? I think it is not the deworming drug that influenced the hemoglobin concertation, rather it may be the supplementations given to the children during the deworming campaign. If this was not the case, I think it is better to remove deworming from the analysis.

**Response:** *The association between deworming and anaemia in very young children has previously been well documented in clinical trials^[4]^*. *Our study showed that for the 329 stool samples tested, none was positive for geohelminth infections. Of these, 285 (86.6%) had received deworming medication during the December 2019 national deworming campaign and 169 (33.9%) were aged 6 to 11 months and at low risk for geohelminth infections. From this analysis we are confident that absence of any parasitic infections from the kato-katz technique in those at risk of infection could be also a result of the deworming campaign. The only additional supplementation received during the campaign was vitamin A which may not explain the association observed in the study.We have recoded the covariate “Deworming” and the analysis now takes into account the very young children (deworming not applicable). We still find a statistically significant relation in the bivariate and the multivariate analysis between anaemia and deworming: adjusted Odd ratio [aOR] of 3.54 and 95% CI 1.79-6.99, p<0.001 for 44 children who didn’t not receive deworming medication last six months out of 329 who were at risk of geohelminth infections (Table 2).*

**Comment 4**: I think the discussion should not be crowded by numbers for comparison. Such

numbers on the prevalence of different study findings shall be presented in the introduction

section. And make the comparisons based on the level of significance and looking your

confidence intervals than putting the numbers. The same thing is true for effect size of the

factors. This comment is misunderstood by the authors. I recommend not to use the numbers which are indicated in the introduction and result section of the manuscript. Remove the odds ratio and its 95% CI in the discussion section.

**Response:** *Thank you for this suggestion. We proceeded as recommended: We presented numbers on the prevalence of different study findings shall be presented in the introduction section (line 45-55, 65-68) and we removed the odds ratios and 95% CI in the discussion section (lines 305, 342-343 and 355).*

1. Elevation map. Elevation map for localities. Elevation of Mukenke, Bwambarangwe, Burundi (Latitude: 2.58 South, Longitude: 29.95 East, Altitude: 1331.00m/4599.74ft and Latitude: 2.58 South, Longitude: 30.32 East, Altitude: 1476.00m/4842.52ft). Elevation map for localities 2021.
2. Centers for Diseases Control and Prevention. CDC criteria for anemia in children and childbearing-aged women. MMWR Morbidity and mortality weekly report. 1989; 38(22):400-4.
3. Sarma H, Wangdi K, Tariqujjaman M, et al. The Effects of Deworming and Multiple Micronutrients on Anaemia in Preschool Children in Bangladesh: Analysis of Five Cross-Sectional Surveys. *Nutrients* 2021; 14(1).
4. Bauleni A, Tiruneh FN, Mwenyenkulu TE, et al. Effects of deworming medication on anaemia among children aged 6-59 months in sub-Saharan Africa. *Parasit Vectors* 2022; 15(1): 7.
